# Supplementary material for: Minimizing Structural Bias in Single-Molecule Super-Resolution Microscopy
Source: Sci Rep. 2018 Sep 3;8:13133. doi: 10.1038/s41598-018-31366-w (PMC6120949; doi:10.1038/s41598-018-31366-w)
Supplement: Supplementary file 1 — Supplementary Info [file 41598_2018_31366_MOESM1_ESM.pdf]

Minimizing Structural Bias in Single-Molecule Super-Resolution Microscopy: Supplementary Information

HESAM MAZIDI[1], **JIN LU**[1], **ARYE NEHORAI**[1], **AND MATTHEW D. LEW**[1*]

[1] *Department of Electrical and Systems Engineering, Washington University in St. Louis, MO 63130, USA*
[*] *Corresponding author: mdlew@wustl.edu*

1. STRUCTURAL ACCURACY AND STRUCTURAL BIAS

Let $\mathscr{T}$ denote the structure of interest that is sufficiently and uniformly labeled by fluorophores. For simplicity, we assume a 1D imaging scenario. Mathematically, we can represent $\mathscr{T}$ as a scalar field defined over a support set $\Omega \in \mathbb{R}$. In particular, we define $\mathscr{T} : \mathbb{R} \to \mathbb{R}$, that is, for each point $\omega \in \Omega$, $\mathscr{T}(\omega)$ represents the labeling density, i.e., the concentration of fluorophores that are activated during the imaging at that point.

Let $\hat{\mathscr{T}}$ represent the structure recovered by any algorithm from a single-molecule localization microscopy dataset (SMLM) $\mathscr{D}$. To statistically quantify the error in recovering $\mathscr{T}$, we define a structural error metric denoted by $\mathscr{R}$ as follows:

$$\mathscr{R}(\hat{\mathscr{T}}, \mathscr{T}) = E_{\mathscr{D}}\Big(l(\mathscr{T} - \hat{\mathscr{T}})\Big), \tag{1.1}$$

where $E_{\mathscr{D}}(\cdot)$ denotes expectation over many SMLM datasets or equivalently a large number of localizations and $l(\cdot)$ represents a loss function. For simplicity, we discretize Ω to obtain a finite set of N object pixels such that $\boldsymbol{\Omega}_d = \{\omega_1, \ldots, \omega_N\}$. Therefore, we can define $\mathscr{T}(\hat{\mathscr{T}})$ over $\boldsymbol{\Omega}_d$ such that $\mathscr{T}_d = [\mathscr{T}_1, \ldots, \mathscr{T}_N]^T (\hat{\mathscr{T}}_d = [\hat{\mathscr{T}}_1, \ldots, \hat{\mathscr{T}}_N]^T)$. We also consider $l(\cdot)$ to be a point-wise square loss, that is, $l(\boldsymbol{e}) = [e_1^2, \ldots, e_N^2]^T$ for $\boldsymbol{e} \in \mathbb{R}^N$. Therefore, we may re-write Eq. 1.1 as follows:

$$\mathscr{R}(\hat{\mathscr{T}}_d, \mathscr{T}_d) = E_{\mathscr{D}}\Big(l(\mathscr{T}_d - \hat{\mathscr{T}}_d)\Big) = E_{\mathscr{D}}\Big(l(\hat{\mathscr{T}}_d - E_{\mathscr{D}}(\hat{\mathscr{T}}_d))\Big) + l\Big((\mathscr{T}_d - E_{\mathscr{D}}(\hat{\mathscr{T}}_d))\Big). \tag{1.2}$$

Note that Eq. 1.2 measures the variation of error over the structure. More importantly, $l\Big((\mathscr{T}_d - E_{\mathscr{D}}(\hat{\mathscr{T}}_d))\Big)$ captures the structure of errors, i.e., the vectorial nature of errors and it measures the statistical inaccuracy in recovering the structure. We refer to $l\Big((\mathscr{T}_d - E_{\mathscr{D}}(\hat{\mathscr{T}}_d))\Big)$ as *structural bias*. This performance characterization differs from conventional analyses in two aspects: (i) the loss function in conventional analyses is defined to be ℓ_2 norm, which collapses $(\mathscr{T}_d - \hat{\mathscr{T}}_d)$ into a scalar value and (ii) the decoupling of errors as in Eq. 1.2 is neglected. We emphasize that in the case of low-density imaging in SMLM in which isolated images of single molecules are analyzed, the *structural accuracy* is guaranteed (statistically), e.g., using maximum likelihood estimation. In other words, $l\Big((\mathscr{T}_d - E_{\mathscr{D}}(\hat{\mathscr{T}}_d))\Big) = 0$.

2. DERIVATION OF THE JOINT MODEL

A. Definitions

Single molecule: in the simplest model, two physical quantities characterize a single molecule (SM): position $\boldsymbol{x} \in \mathbb{R}^3$ and brightness s measured as the number of detected photons. Mathematically, it is equivalent to an impulse located at $\boldsymbol{x} \in \mathbb{R}^3$ with magnitude of s: $s\delta(\boldsymbol{x})$.
Object space: any physical distribution of molecules may be described by a scalar function $f : \mathbb{R}^3 \mapsto \mathbb{R}$. For example, the distribution of three single molecules located at $\boldsymbol{x}_1, \boldsymbol{x}_2$, and $\boldsymbol{x}_3$ with brightnesses s_1, s_2, and s_3, respectively, is described by $f(\boldsymbol{x}) = \sum_{i=1}^{3} s_i \delta(\boldsymbol{x} - \boldsymbol{x}_i)$. The set of all functions describing the distribution of single molecules is called the object space Ξ.
Image space: the set of all functions $g : \mathbb{R}^2 \mapsto \mathbb{R}$ describing the output images of the imaging system is called the image space $\mathscr{I}$.
Point spread function and imaging operator: a linear shift-invariant imaging system is characterized by the so-called point spread function (PSF) $h : \mathbb{R}^2 \mapsto \mathbb{R}$, which completely describes a continuous convolution operator mapping the object space into the image space. The PSF of the imaging system captures the propagation and modulation of the light from a single molecule to the camera. We call this continuous convolution operator associated with h as the imaging operator $Q_{h_\theta} : \Xi \mapsto \mathscr{I}$ (θ designates a set of parameters such as depth):

$$g(\boldsymbol{u}) = Q_{h_\theta}(f) = \int_{-\infty}^{+\infty} h_\theta(\boldsymbol{u} - \boldsymbol{x}) f(\boldsymbol{x})\, d\boldsymbol{x}, \quad \boldsymbol{u} \in \mathbb{R}^2, f \in \Xi, g \in \mathscr{I}. \tag{2.1}$$

We consider a finite collection of molecules, $f(\boldsymbol{x}) = \sum_{i=1}^{N} s_i \delta(\boldsymbol{x} - \boldsymbol{x}_i)$, so that Eq. (2.1) reduces to

$$g(\boldsymbol{u}) = Q_{h_\theta}(f) = \sum_{i=1}^{N} s_i h_\theta(\boldsymbol{u} - \boldsymbol{x}_i), \quad \boldsymbol{u} \in \mathbb{R}^2, g \in \mathscr{I}. \tag{2.2}$$

Our goal is to rigorously define the approximations we make to obtain the joint model discussed in the main text. Without loss of generality, we focus on 1D imaging scenario.

B. First-order approximation

Let $D \subset \mathbb{R} = \{d_i = i \times 2r \mid i \in \{-\mathscr{N}, -\mathscr{N}+1, \ldots, 0, \ldots, \mathscr{N}\}, \quad r > 0\}$ be a set of discrete points in $\mathbb{R}$ called the grid points. For all positions $x \in [-\mathscr{N}2r, \mathscr{N}2r]$ we can uniquely define $x = d + \delta$ for some appropriate choice of $\delta \in [-r, r)$ and $d \in D$. Therefore, we can define the object space restricted to $x \in [-\mathscr{N}2r, \mathscr{N}2r]$ in terms of D and $\delta \in [-r, r)$. In particular for $f(x) = \sum_{i=1}^{N} s_i \delta(x - x_i)$, we have that $f(x) = \sum_{i=1}^{N} s_i \delta(x - d_i - \delta_i)$, where it is understood that $x_i = d_i + \delta_i$ for some $d_i \in D$ and $i = 1 : N$.

With this new representation of the object space at hand, we may re-write Eq. (2.2) as follows:

$$g(u) = Q_{h_\theta}(f) = \sum_{i=1}^{N} s_i h_\theta(u - d_i - \delta_i), \quad u \in \mathbb{R}, f \in \Xi, g \in \mathscr{I}. \tag{2.3}$$

For a fixed $i \in \{1, \cdots, N\}$ we use the Taylor expansion of $h_\theta(\cdot)$ as follows:

$$h_\theta(u - d_i - \delta_i) = h_\theta(u - d_i) - h'_\theta(u - d_i)\delta_i + k(u - d_i; \delta_i),$$

where $h'_\theta(\cdot)$ is the derivative of $h_\theta(\cdot)$ and $k(\cdot;\cdot)$ denotes the remainder term in the Taylor expansion of $h_\theta(\cdot)$. We keep the first-order term and neglect the remainder term to obtain the first-order approximation of $h_\theta(\cdot)$ denoted by $\hat{h}_\theta(\cdot)$:

$$\hat{h}_\theta(u - d_i - \delta_i) = h_\theta(u - d_i) - h'_\theta(u - d_i)\delta_i. \tag{2.4}$$

Using Eq. (2.4), we obtain an approximation of $g(\cdot)$:

$$\begin{aligned} g(u) = Q_{\hat{h}_\theta}(f) &= \sum_{i=1}^{N} s_i \Big(h_\theta(u - d_i) - h'_\theta(u - d_i)\delta_i \Big) \\ &= \sum_{i=1}^{N} s_i h_\theta(u - d_i) - \sum_{i=1}^{N} s_i h'_\theta(u - d_i)\delta_i, \end{aligned} \tag{2.5}$$

where $u \in \mathbb{R}, f \in \Xi$, and $g \in \mathscr{I}$. By letting $p_i = -\delta_i s_i$ and $q(\cdot) = h'_\theta(\cdot)$, we have:

$$g(u) = Q_{h_\theta}(f_{\boldsymbol{d},\boldsymbol{s}}) + Q_q(f_{\boldsymbol{d},\boldsymbol{p}}), \tag{2.6}$$

where $f_{\boldsymbol{d},\boldsymbol{s}}(x) = \sum_{i=1}^{N} s_i \delta(x - d_i)$, $f_{\boldsymbol{d},\boldsymbol{p}}(x) = \sum_{i=1}^{N} p_i \delta(x - d_i)$, $\boldsymbol{d} = [d_1, \ldots, d_N]^T$, $\boldsymbol{s} = [s_1, \ldots, s_N]^T$, and $\boldsymbol{p} = [p_1, \ldots, p_N]^T$. We note that $-s_i r \le p_i < s_i r$ for $i = 1 : N$. It is apparent from Eq. (2.6) that $\hat{g}(u)$ is sum of two functions that are obtained through a continuous convolution operator with different PSFs.

We define a first-order approximation operator $\hat{Q} : \mathscr{I} \mapsto \mathscr{I}$ as follows:

$$g(u) = \hat{Q}(g) = \hat{Q}\Big(Q_{h_\theta}(f)\Big) = Q_{h_\theta}(f_{\boldsymbol{d},\boldsymbol{s}}) + Q_q(f_{\boldsymbol{d},\boldsymbol{p}}),$$

where $u \in \mathbb{R}, f \in \Xi, g \in \mathscr{I}$. The key observation here is that the first-order approximation operator along with the imaging operator define two imaging operators with different PSFs and input functions. This observation becomes very useful in implementing the algorithm efficiently using unique properties of convolution (section 2).

C. Camera integration as a linear operator

Let $I : \mathscr{I} \mapsto \mathscr{I}_m$ denote an integration operator where $\mathscr{I}_m$ is the space of vectors of length m. Concretely, let C_i be the integration interval of the i_{th} pixel of the camera. We then have the following characterization for I:

$$\boldsymbol{g} = I(g) = [\int_{C_1} g(u)\, du, \cdots, \int_{C_m} g(u)\, du]^T. \tag{2.7}$$

Clearly, I is a linear operator:

$$\begin{aligned} I(\alpha_1 g_1 + \alpha_2 g_2) &= [\int_{C_1} \alpha_1 g_1(u) + \alpha_2 g_2(u)\, du, \cdots, \int_{C_m} \alpha_1 g_1(u) + \alpha_2 g_2(u)\, du]^T \\ &= \alpha_1 I(g_1) + \alpha_2 I(g_2). \end{aligned} \tag{2.8}$$

We can now describe our joint imaging model through three operators, namely imaging operator Q_{h_θ}, the first- order approximation operator $\hat{Q}$, and the integration operator I:

$$\boldsymbol{g} = I\Big(\hat{Q}\Big(Q_{h_\theta}(f)\Big)\Big). \tag{2.9}$$

D. Putting it all together

For a finite collection of molecules we can conveniently write Eq. (2.9) equivalently in a matrix-vector notation. Plugging Eq. (2.6) into Eq. (2.7) we have that:

$$g_j = \sum_{i=1}^{N} s_i \int_{C_j} h(u - d_i)\, du + \sum_{i=1}^{N} p_i \int_{C_j} q(u - d_i)\, du, \; j = 1 : m. \tag{2.10}$$

Define $\Phi_{i,j} = \int_{C_j} h(u - d_i)\, du$ and $G_{i,j} = \int_{C_j} q(u - d_i)\, du$ for $i = 1 : N, j = 1 : m$. Further, let $\boldsymbol{\gamma} = [s_1, \ldots, s_N, p_1, \ldots, p_N]^T$. Then we have

$$\boldsymbol{g} = \boldsymbol{A}\boldsymbol{\gamma}, \quad \boldsymbol{\gamma} \in \mathscr{C}, \tag{2.11}$$

where $\boldsymbol{A} = [\boldsymbol{\Phi}\ \boldsymbol{G}]$ and $\mathscr{C} = \{s_i \ge 0, \quad -s_i r \le p_i < s_i r, \quad i = 1 : N\}$. This completes the derivation of the joint model in 1D within the first-order approximation. The extension to 3D follows immediately:

$$\begin{aligned} &\boldsymbol{A} = [\boldsymbol{\Phi}, \boldsymbol{G}_x \boldsymbol{G}_y, \boldsymbol{G}_z], \\ &\boldsymbol{\gamma} = [\boldsymbol{s}^T, \boldsymbol{s}^T \odot \boldsymbol{\Delta x}^T, \boldsymbol{s}^T \odot \boldsymbol{\Delta y}^T, \boldsymbol{s}^T \odot \boldsymbol{\Delta z}^T]^T \\ &\quad = [\boldsymbol{s}^T, \boldsymbol{p}_x^T, \boldsymbol{p}_y^T, \boldsymbol{p}_z^T], \\ &\mathscr{C} = \{s_i \ge 0, \quad -s_i r_j \le p_{j,i} < s_i r_j, \quad i = 1 : N, j \in \{x, y, z\}\} \end{aligned} \tag{2.12}$$

We would like to stress that our derivation of joint model applies to an arbitrary PSF.

In many imaging problems, including fluorescence microscopy, Poisson-distributed shot noise dominates the photon-measurement process. The measured image at i_{th} pixel, g_i, on the camera follows a Poisson distribution as follows:

$$g_i \sim \mathscr{P}\Big((\boldsymbol{A}\boldsymbol{\gamma})_i + b_i\Big), \tag{2.13}$$

where $\mathscr{P}$ denotes the Poisson distribution and b_i represents a positive background at pixel $i \in \{1,\ldots,m\}$. Assuming that image pixels are statistically independent, we may write the statistical image model for the measured image $\boldsymbol{g}$ as a Poissonian probability density:

$$p(\boldsymbol{g}|\boldsymbol{A}\boldsymbol{\gamma}+\boldsymbol{b}) = \prod_{i=1}^{m} \frac{[(\boldsymbol{A}\boldsymbol{\gamma}+b)_i]^{g_i}\, e^{-(\boldsymbol{A}\boldsymbol{\gamma}+b)_i}}{g_i!}. \tag{2.14}$$

The negative Poisson log-likelihood for the model in Eq. (2.14) (neglecting constant terms) is given by:

$$\mathscr{L}(\boldsymbol{\gamma};\boldsymbol{g},\boldsymbol{A},\boldsymbol{b}) = \boldsymbol{1}^T(\boldsymbol{A}\boldsymbol{\gamma}+\boldsymbol{b}) - \sum_{i=1}^{m} \log\Big((\boldsymbol{A}\boldsymbol{\gamma}+\boldsymbol{b})_i\Big) g_i. \tag{2.15}$$

Throughout our analysis we assume that an estimate of the background is given.

3. MATHEMATICAL DESCRIPTION OF RoSE

A. identifying single molecules via structured deconvolution

The structured deconvolution can be cast as follows:

$$\min_{\boldsymbol{\gamma}\in\mathscr{C}} \mathscr{L}(\boldsymbol{\gamma};\boldsymbol{g},\boldsymbol{A},\boldsymbol{b}) + \lambda\|\boldsymbol{\gamma}\|_{1,2}, \tag{3.1}$$

where $\|\cdot\|_{1,2}$ denotes the mixed $\ell_{1,2}$ norm to enforce joint sparsity in $\boldsymbol{\gamma}$ and λ is a penalty parameter. In particular, the mixed $\ell_{1,2}$ is defined as:

$$\|\boldsymbol{\gamma}\|_{1,2} = \sum_{i=1}^{N} \sqrt{s_i^2 + p_{x,i}^2 + p_{y,i}^2 + p_{z,i}^2}.$$

In order to show that Eq. (3.1) is a convex program, it remains to show that the Poisson log-likelihood is convex over its domain (which is convex): $\{\boldsymbol{\gamma}\in\mathbb{R}^N, \boldsymbol{b}\in\mathbb{R}^m | \boldsymbol{A}\boldsymbol{\gamma}+\boldsymbol{b} > 0\}$, where we assume that $b_i > 0$ for $i = 1:m$.

In the following section we prove the joint convexity of the $\mathscr{L}$ with respect to $\boldsymbol{\gamma}$ and $\boldsymbol{b}$. First, we notice the following:

$$\nabla_{\boldsymbol{\gamma}}\mathscr{L} = -(\boldsymbol{1}^T\boldsymbol{A})^T - \boldsymbol{A}^T \mathsf{diag}\Big(\frac{\boldsymbol{1}}{\boldsymbol{A}\boldsymbol{\gamma}+\boldsymbol{b}}\Big)\boldsymbol{g}, \tag{3.2}$$

where $\boldsymbol{1} = [1\ldots1]^T \in \mathbb{R}^m$. Therefore,

$$\nabla^2_{\boldsymbol{\gamma}}\mathscr{L} = \boldsymbol{A}^T \mathsf{diag}\Big(\boldsymbol{g}\odot\frac{\boldsymbol{1}}{(\boldsymbol{A}\boldsymbol{\gamma}+\boldsymbol{b})^2}\Big)\boldsymbol{A}. \tag{3.3}$$

Similarly, we have

$$\nabla_{\boldsymbol{b}}\mathscr{L} = \boldsymbol{1} - \mathsf{diag}\Big(\frac{\boldsymbol{1}}{\boldsymbol{A}\boldsymbol{\gamma}+\boldsymbol{b}}\Big)\boldsymbol{g}, \qquad \nabla^2_{\boldsymbol{b}}\mathscr{L} = \mathsf{diag}\Big(\boldsymbol{g}\odot\frac{\boldsymbol{1}}{(\boldsymbol{A}\boldsymbol{\gamma}+\boldsymbol{b})^2}\Big),$$

$$\nabla_{\boldsymbol{b}\boldsymbol{\gamma}}\mathscr{L} = \mathsf{diag}\Big(\boldsymbol{g}\odot\frac{\boldsymbol{1}}{(\boldsymbol{A}\boldsymbol{\gamma}+\boldsymbol{b})^2}\Big)\boldsymbol{A}.$$

Let $\boldsymbol{D} \triangleq \mathsf{diag}\big(\boldsymbol{g}\odot\frac{\boldsymbol{1}}{(\boldsymbol{A}\boldsymbol{\gamma}+\boldsymbol{b})^2}\big)$. Then we can write the Hessian of $\mathscr{L}$ as follows:

$$\nabla^2\mathscr{L} = \begin{bmatrix} \nabla^2_{\boldsymbol{\gamma}}\mathscr{L} & \nabla_{\boldsymbol{b}\boldsymbol{\gamma}}\mathscr{L} \\ \nabla_{\boldsymbol{b}\boldsymbol{\gamma}}\mathscr{L} & \nabla^2_{\boldsymbol{b}}\mathscr{L} \end{bmatrix} = \begin{bmatrix} \boldsymbol{A}^T\boldsymbol{D}\boldsymbol{A} & \boldsymbol{A}^T\boldsymbol{D} \\ \boldsymbol{D}\boldsymbol{A} & \boldsymbol{D} \end{bmatrix}$$

We note that $\boldsymbol{D} > 0$ since $\boldsymbol{A}\boldsymbol{\gamma}+\boldsymbol{b} > 0$. Then clearly $\boldsymbol{D} > 0$ and we only need to show the following [1]:

$$\boldsymbol{A}^T\boldsymbol{D}\boldsymbol{A} - \boldsymbol{A}^T\boldsymbol{D}\boldsymbol{D}^{-1}\boldsymbol{D}\boldsymbol{A} \succeq 0, \tag{3.4}$$

which is trivially satisfied. It follows that $\nabla^2\mathscr{L} \geq 0$ and thus $\mathscr{L}$ is convex over $\{\boldsymbol{\gamma}\in\mathbb{R}^N, \boldsymbol{b}\in\mathbb{R}^M | \boldsymbol{A}\boldsymbol{\gamma}+\boldsymbol{b} > 0\}$.

To solve Eq. (3.1) we proceed by transforming Eq. (3.1) into an unconstrained optimization:

$$\min_{\boldsymbol{\gamma}} \mathscr{L}(\boldsymbol{\gamma};\boldsymbol{g},\boldsymbol{A},\boldsymbol{b}) + \lambda\|\boldsymbol{\gamma}\|_{1,2} + I_{\mathscr{C}}(\boldsymbol{\gamma}), \tag{3.5}$$

where $I_{\mathscr{C}}(\cdot)$ is the indicator function of $\mathscr{C}$:

$$I_{\mathscr{C}}(\boldsymbol{\gamma}) = \begin{cases} 0 & \gamma\in\mathscr{C} \\ +\infty & \gamma\notin\mathscr{C}. \end{cases}$$

Solving Eq. (3.5) using accelerated gradient algorithms requires the proximal operator of $\left(\lambda\|\boldsymbol{\gamma}\|_{1,2}+I_{\mathscr{C}}(\boldsymbol{\gamma})\right)$, which does not admit a closed-form solution [2]. To tackle this, we utilize the smoothing technique to approximate $w(\gamma)=\lambda\|\boldsymbol{\gamma}\|_{1,2}$ with a differentiable function, e.g., its Moreau envelope [3], and proceed to compute the proximal operator of $I_{\mathscr{C}}(\boldsymbol{\gamma})$. The Moreau envelope of $w(\cdot)$ is continuously differentiable, and its gradient is given by:

$$\nabla w_\mu(\boldsymbol{\gamma})=\frac{1}{\mu}(\boldsymbol{\gamma}-\mathrm{prox}_{\mu w}(\boldsymbol{\gamma})),$$

where $\mathrm{prox}_{\mu w}(\cdot)$ denotes the proximal operator of $\mu w(\cdot)$. Note that μ is the smoothing parameter, which controls the accuracy of approximation. Importantly, the Moreau envelope has a Lipschitz constant of $1/\mu$.

The proximal operator of $I_{\mathscr{C}}(\cdot)$ is given by:

$$\mathrm{prox}_{I_{\mathscr{C}}}(\boldsymbol{\gamma})=P_{\mathscr{C}}(\boldsymbol{\gamma}), \tag{3.6}$$

where $P_{\mathscr{C}}(\cdot)$ is the projection operator onto the set $\mathscr{C}$. Noting that $\mathscr{C}$ is the intersection of sets $\mathscr{C}=\bigcap_{i=1}^{N}\mathscr{C}_i=\{s_i\geq 0,\quad -s_ir_j\leq p_{j,i}<s_ir_j,\quad j\in\{x,y,z\}\}$, we can compute Eq. (3.6) element-wise as:

$$\mathrm{prox}_{\mathscr{I}_{\mathscr{C}}}(\gamma_i=[s_i,p_{x,i},p_{y,i},p_{z,i}]^T)=P_{\mathscr{C}_i}(\gamma_i). \tag{3.7}$$

Note that for $r=1$, $\mathscr{C}_i=\{\gamma_i|\ \|[p_{x,i},p_{y,i},p_{z,i}]^T\|_\infty\leq s_i\}$, which is the norm cone associated with $\|\cdot\|_\infty$. Unfortunately, the element-wise projection operator in Eq. (3.7) does not have a closed-form expression. Interestingly, we can approximate the set $\mathscr{C}_i$ with a second order cone $\widehat{\mathscr{C}_i}$:

$$\widehat{\mathscr{C}_i}=\{\gamma_i|\ \|[p_{x,i},p_{y,i},p_{z,i}]^T\|_2\leq rs_i\}, \tag{3.8}$$

where we assume for simplicity $r_x=r_y=r_z=r$. This approximation allows us to obtain a *closed-form* expression for the element-wise projection operator:

$$P_{\widehat{\mathscr{C}_i}}(\gamma_i)=\begin{cases}0 & \|\boldsymbol{p}_i\|_2\leq -s_i/r\\ (s_i,\boldsymbol{p}) & \|\boldsymbol{p}_i\|_2\leq s_ir\\ (\frac{s_i+r\|\boldsymbol{p}\|_2}{1+r^2},\frac{s_i+r\|\boldsymbol{p}\|_2}{1+r^2}\cdot\frac{r}{\|\boldsymbol{p}\|_2}\boldsymbol{p}) & \|\boldsymbol{p}_i\|_2>s_ir,\end{cases} \tag{3.9}$$

where $\boldsymbol{p}=(p_{x,i},p_{y,i},p_{z,i})$.

Although selecting the step size as the inverse of Lipschitz constant of the objective function guarantees convergence of an accelerated gradient algorithm [4], the speed with which it converges hinges upon the careful choice of this step size. Moreover, the Lipschitz constant of the Poisson negative log-likelihood $\mathscr{L}(\boldsymbol{\gamma};\boldsymbol{g},\boldsymbol{A},\boldsymbol{b})$ is unknown, which poses a challenge for robust implementation of the algorithm. To tackle this, we employ an adaptive strategy to ensure robustness of the algorithm and its fast convergence. In particular, we first estimate an initial step size by computing an upper bound on the Lipschitz constant of the Poisson negative log-likelihood (L_u) [5]. We then utilize the backtracking technique to ensure the convergence of the algorithm. The resulting structured deconvolution program is summarized in algorithm S1.

Algorithm S1. Structured deconvolution program

Input: $\{\boldsymbol{g},\boldsymbol{\gamma}_0,\boldsymbol{b},\mu,\lambda,L_u\}$
Step 0. Take $\boldsymbol{v}_1=\boldsymbol{\gamma}_0, t_1=1$, and $\eta>1$. Set $L_0=L_u/L_t$. ▷ L_t denotes a constant chosen to initialize the inverse of the step size.
Step k. $(k\geq 1)$ Find the smallest integer $i_k>0$ such that with $\overline{L}=\eta^{i_k}L_{k-1}$:
$\mathscr{L}(\overline{\boldsymbol{v}}_k^{\overline{L}})\leq\mathscr{L}(\boldsymbol{v}_{\boldsymbol{k}})+[\nabla_{\boldsymbol{v}}\mathscr{L}(\boldsymbol{v}_k)+\nabla w_\mu(\boldsymbol{v}_k)]^T[\overline{\boldsymbol{v}}_k^{\overline{L}}-\boldsymbol{v}_{\boldsymbol{k}}]+\overline{L}\|v_k-\overline{\boldsymbol{v}}_k^{\overline{L}}\|_2^2$ ▷ $\overline{\boldsymbol{v}}_k^{\overline{L}}=P_{\widehat{\mathscr{C}}}(\boldsymbol{v}_k-\frac{1}{\overline{L}}[\nabla_{\boldsymbol{v}}\mathscr{L}(\boldsymbol{v}_k)+\nabla w_\mu(\boldsymbol{v}_k)])$
Set $L_k=\eta^{i_k}L_{k-1}$ and Compute:
    $\nabla_{\boldsymbol{v}}\mathscr{L}(\boldsymbol{v}_k)=-(\boldsymbol{1}^T\boldsymbol{A})^T-\boldsymbol{A}^T\mathsf{diag}(\frac{1}{\boldsymbol{A}\boldsymbol{v}_k+\boldsymbol{b}})\boldsymbol{g}$,
    $\nabla w_\mu(\boldsymbol{v}_k)=\frac{1}{\mu}(\boldsymbol{v}_k-\mathrm{prox}_{\mu w}(\boldsymbol{v}_k))$,
    $\boldsymbol{\gamma}_k=P_{\widehat{\mathscr{C}}}(\boldsymbol{v}_k-\frac{1}{L_k}[\nabla_{\boldsymbol{v}}\mathscr{L}(\boldsymbol{v}_k)+\nabla w_\mu(\boldsymbol{v}_k)])$,
    $t_k=\frac{1+\sqrt{1+4t_k^2}}{2}$,
    $\boldsymbol{v}_{k+1}=\boldsymbol{\gamma}_k+\frac{t_k-1}{t_{k+1}}(\boldsymbol{\gamma}_k-\boldsymbol{\gamma}_{k-1})$.

In order to efficiently compute the update steps in algorithm S1 we describe how to leverage the properties of convolution operator. From Eq. 2.5, which represents the imaging model in 1D, we have:

$$g(u)=\sum_{i=1}^{N}s_ih_\theta(u-d_i)-\sum_{i=1}^{N}s_ih'_\theta(u-d_i)\delta_i, \tag{3.10}$$

which consists of two distinct discrete convolution operations. By assuming *periodic end conditions* for $\boldsymbol{s}$ and $\boldsymbol{p}$, one can show that Eq. 3.10 becomes sum of two N-point *circulant* convolutions. Assuming same pixelation on the camera as the object space, i.e., $m=N$, we can re-write these convolution operations in a matrix-vector form according to Eq. 2.11:

$$\boldsymbol{g}=\boldsymbol{\Phi}\boldsymbol{s}+\boldsymbol{G}\boldsymbol{p}, \tag{3.11}$$

where $\boldsymbol{\Phi}\in\mathbb{R}^{N\times N}$ and $\boldsymbol{G}\in\mathbb{R}^{N\times N}$ are now circulant matrices. Without loss of generality, we focus on computing terms involving $\boldsymbol{\Phi}$. It turns out that $\boldsymbol{\Phi}=\frac{1}{N}\mathscr{Q}^H\Lambda\mathscr{Q}$ in which $\mathscr{Q}$ is the $N\times N$ discrete Fourier transform (DFT) matrix, $\mathscr{Q}^H$ is the Hermitian transpose of $\mathscr{Q}$, and Λ is a diagonal matrix

with elements equal to the DFT of the sampled PSF, i.e., $[h_\theta(-N/2-1),\dots,h_\theta(N/2)]^T$. The computation of gradient of the Poisson negative log-likelihood in algorithm S1 involves two major matrix-vector multiplications, i.e., $\boldsymbol{Av}$ and $\boldsymbol{A}^T\tilde{\boldsymbol{g}}$. First, notice that $\boldsymbol{Av}=\boldsymbol{\Phi s}+\boldsymbol{Gp}$. From above discussion, we have $\boldsymbol{\Phi s}=\frac{1}{N}\mathscr{Q}^H\Lambda\mathscr{Q}\boldsymbol{s}$ which can be efficiently computed by noting that $\mathscr{Q}\boldsymbol{s}$ is essentially the DFT of $\boldsymbol{s}$ followed by an element-wise multiplication with Λ in frequency domain and performing an inverse DFT, resulting in a computational complexity of $O(N\log(N))$. Similarly, $\boldsymbol{A}^T\tilde{\boldsymbol{g}}=[(\boldsymbol{\Phi}^T\tilde{\boldsymbol{g}})^T;(\boldsymbol{G}^T\tilde{\boldsymbol{g}})^T]^T$ and that $\boldsymbol{\Phi}^T\tilde{\boldsymbol{g}}=(\frac{1}{N}\mathscr{Q}^H\Lambda\mathscr{Q})^H\boldsymbol{g}=\frac{1}{N}\mathscr{Q}^H\Lambda^H\mathscr{Q}\boldsymbol{g}$, which has the same computational complexity of $O(N\log(N))$.

Similar arguments hold for computing matrix-vector products involving $\boldsymbol{G}$. In fact, this extends to 2D and 3D models involving matrices $\boldsymbol{G}_x,\boldsymbol{G}_y$ and $\boldsymbol{G}_z$, which significantly reduces the complexity of the algorithm in terms of both memory and computation, especially over a large field of view.

B. Construction of GradMap

In order to demix precise position estimates from brightness estimates we leverage the joint recovery of molecular parameters by algorithm S1. In particular, recovered position gradients converge to precise positions of molecular blinking events. This observation allows us to compute a tensor, called GradMap $\mathscr{G}$, in which $\mathscr{G}(i)$ represents the degree of convergence to i_{th} grid point. To construct $\mathscr{G}$ at each grid point i from output of algorithm S1 denoted by $\boldsymbol{\gamma}_{\text{f}}=[s_{\text{f}},\boldsymbol{p}_{\text{f}}]^T$, we first obtain initial estimates of molecular parameters $(\hat{s}_i,\widehat{\Delta x}_i,\widehat{\Delta y}_i,\widehat{\Delta z}_i)$ as follows:

$$
\begin{aligned}
\hat{s}_i &= s_{\text{f},i}\cdot I_{s_{\text{f},i}\geq s_0}(s_{\text{f},i}),\\
\widehat{\Delta x}_i &= \frac{p_{x,\text{f},i}}{s_{\text{f},i}}\cdot I_{s_{\text{f},i}\geq s_0}(s_{\text{f},i}),\\
\widehat{\Delta y}_i &= \frac{p_{y,\text{f},i}}{s_{\text{f},i}}\cdot I_{s_{\text{f},i}\geq s_0}(s_{\text{f},i}),\\
\widehat{\Delta z}_i &= \frac{p_{z,\text{f},i}}{s_{\text{f},i}}\cdot I_{s_{\text{f},i}\geq s_0}(s_{\text{f},i}),\quad i\in\{1,\dots,N\},
\end{aligned}
$$

where s_0 is a small value relative to maximum value of the recovered brightnesses.

Let N_i be a set of grid points surrounding the grid-point i (e.g., in 2D, N_i contains 8 points). Define the source coefficient, $\mathscr{G}(i)$, associated with i_{th} grid point located at $[x_{g,i},y_{g,i},z_{g,i}]^T$ as follows:

$$
\alpha_{ji}=\frac{[x_{g,i}-x_{g,j},y_{g,i}-y_{g,j},z_{g,i}-z_{g,j}]\;[\widehat{\Delta x}_j,\widehat{\Delta y}_j,\widehat{\Delta z}_j]^T}{\|[x_{g,i}-x_{g,j},y_{g,i}-y_{g,j},z_{g,i}-z_{g,j}]^T\|_2\;\|[\widehat{\Delta x}_j,\widehat{\Delta y}_j,\widehat{\Delta z}_j]^T\|_2},\qquad j\in N_i, \tag{3.12}
$$

$$
\mathscr{G}(i)=\frac{\sum_{j\in N_i}\hat{s}_j\alpha_{ji}}{\sum_{j\in N_i}\hat{s}_i}. \tag{3.13}
$$

Note that α_{ji} describes convergence of the position gradients from j_{th} grid point to i_{th} grid point and $\mathscr{G}(i)\in[-1,1]$ measures the local degree of convergence corresponding to i_{th} grid point. Consequently, the number of activated molecules is obtained from the local maxima of the GradMap and their initial parameters are calculated by averaging the molecular parameters in the corresponding neighborhoods.

C. Adaptive constrained maximum likelihood

Let's denote the initial estimates by γ_{init}. We define the support of γ as $\text{Supp}(\gamma)=\{i\mid s_i>0,i\in\{1,\dots,N\}\}$. For accurate and precise recovery, it is crucial to design a robust debiasing algorithm to minimize $|\boldsymbol{\gamma}_{\text{init}}-\boldsymbol{\gamma}_{\text{true}}|$, especially when $\text{Supp}(\boldsymbol{\gamma}_{\text{init}})$ differs from the true support by more than a few grid points. To tackle this task, we consider a constrained maximum likelihood for which at each iteration $\text{Supp}(\boldsymbol{\gamma})$ is updated such that molecules are matched to their closest grid points. This adaptive maximum likelihood is summarized in algorithm S2.

Algorithm S2. Adaptive maximum likelihood

Input: $\{\boldsymbol{g},\boldsymbol{\gamma}_{\text{init}},\boldsymbol{b},L_{\text{init}}\}$ ▷ L_{init} is the estimate of the Lipschitz constant obtained from algorithm S1
Step 0. Take $\boldsymbol{v}_1=\boldsymbol{\gamma}_{\text{init}},t_1=1,$ and $\eta>1$. Set $L_0=L_{\text{init}}/L_t$.
Step k. $(k\geq 1)$ Find the smallest integer $i_k>0$ such that with $\bar{L}=\eta^{i_k}L_{k-1}$:
$\mathscr{L}(\bar{v}_k^L)\leq\mathscr{L}(v_k)+[\nabla_{\boldsymbol{v}}\mathscr{L}(\boldsymbol{v}_k)]^T[\bar{v}_k^L-v_k]+\bar{L}\|v_k-\bar{v}_k^L\|_2^2$ ▷ $\bar{v}_k^L=P_{\text{Supp}(\boldsymbol{\gamma}_k)\cap\hat{\mathscr{C}}}(v_k-\frac{1}{\bar{L}}[\nabla_{\boldsymbol{v}}\mathscr{L}(\boldsymbol{v}_k)])$
Set $L_k=\eta^{i_k}L_{k-1}$ and compute:
$\nabla_{\boldsymbol{v}}\mathscr{L}(\boldsymbol{v}_k)=-(\boldsymbol{1}^T\boldsymbol{A})^T-\boldsymbol{A}^T\text{diag}(\frac{1}{\boldsymbol{Av}_k+\boldsymbol{b}})\boldsymbol{g}$,
$\boldsymbol{\gamma}_k=P_{\text{Supp}(\boldsymbol{\gamma}_k)\cap\hat{\mathscr{C}}}(\boldsymbol{v}_k-\frac{1}{L_k}\nabla_{\boldsymbol{v}}\mathscr{L}(\boldsymbol{v}_k))$,
$t_k=\frac{1+\sqrt{1+4t_k^2}}{2}$,
$\boldsymbol{v}_{k+1}=\boldsymbol{\gamma}_k+\frac{t_k-1}{t_{k+1}}(\boldsymbol{\gamma}_k-\boldsymbol{\gamma}_{k-1})$.
if DistSupp$(\boldsymbol{\gamma}_k)\neq$ Supp$(\boldsymbol{\gamma}_k)$ **then** ▷ DistSupp computes the support of $\boldsymbol{\gamma}_k$ based on a minimum distance to the grid points
    UpdateSupp$(\boldsymbol{\gamma}_k)$ ▷ UpdateSupp updates the support of $\boldsymbol{\gamma}_k$ so that every molecule matches it closest grid point

D. Exploiting temporal blinking statistics via GradMap

Let's denote the GradMap estimated at t_{th} frame by $\mathscr{G}_t$ and assume we have T frames in total. We may think of $\mathscr{G}_t(i),i\in\{1,\dots,N\}$, as the probability of a molecule being activated in a small neighborhood of i_{th} grid point. In general, molecules that sample the structure within such a small neighborhood may be activated multiple times during T frames. Therefore, the spatial information regarding the structure, encoded into

$\mathscr{G}$, is correlated over time. Equivalently, if the structure is labeled with enough molecular density such that within a small neighborhood multiple molecules stochastically fluoresce even once, the same structural information captured by $\mathscr{G}$ is correlated over time.

To leverage this correlated structural information, we apply a pixel-wise temporal autocorrelation [6] on the stack of GradMap to obtain a correlated GradMap $\overline{\mathscr{G}_c}$:

$$\mathscr{G}_c = \sum_{t_1}^{T} \sum_{t_2=t_1}^{T} \mathscr{G}_{t_1} \odot \mathscr{G}_{t_2} \tag{3.14}$$

$$\overline{\mathscr{G}_c} = \frac{\mathscr{G}_c}{\max(\mathscr{G}_c)} \tag{3.15}$$

Interestingly, we may think of $\overline{\mathscr{G}_c}$ as the probability that i_{th} pixel represents the structure. Put it differently, $\overline{\mathscr{G}_c}$ assigns a *confidence* level to each localization that signifies its uncertainty in representing the ground-truth structure. Based on this observation, RoSE-C eliminates the localizations such that the corresponding values in $\overline{\mathscr{G}_c}$ are smaller than a threshold, which represents our tolerance regarding the structural confidence level.

We note that the optimal order of temporal correlation is affected by the underling localization precision. In particular, for low SBR levels such that the FWHM of localization precision is comparable to grid size we apply a third-order temporal autocorrelation to minimize the effect of Poisson shot noise.

Given a uniform labeling density and sampling rate, we can set the threshold to be a small constant value. However, in a real experiment, factors such as non-uniform labeling density and sampling rate complicate the choice of threshold value. To tackle these challenges, we utilize simple statistics such as median and average of the correlated GradMap to obtain robustness. First, we normalize the correlated GradMap by only considering typical pixels. To this end, we define the median absolute deviation (MAD) of the correlated GradMap $\mathscr{G}_c$ as follows:

$$MAD = \text{median}(|\mathscr{G}_c(i) - \text{median}(\mathscr{G}_c)|), \tag{3.16}$$

where $i \in \{1, \ldots, N\}$. We define typical pixels of $\mathscr{G}_c$ as follows:

$$T_{\mathscr{G}_c} = \{i \in \{1, \ldots, N\} | \mathscr{G}_c(i) < 50\, MAD\}. \tag{3.17}$$

Accordingly, we normalize $\mathscr{G}_c$ such that $\overline{\mathscr{G}_c} = \frac{\mathscr{G}_c}{\max(\mathscr{G}_c(T_{\mathscr{G}_c}))}$. Finally, we set the confidence level of pixel i with $\mathscr{G}_c(i) \geq 50\, MAD$ exhibiting much larger auto-correlation to 1. Next, we compute the threshold of RoSE-C based on the average confidence levels of all pixels with confidence greater than 0.1:

$$\text{thres} = 0.1\ \text{mean}(\overline{\mathscr{G}_c} > 0.1). \tag{3.18}$$

E. Computing the regularizer

To obtain a robust strategy for computing λ in algorithm S1 we first consider 3.1 :

$$\min_{\boldsymbol{\gamma} \in \mathscr{C}} \mathscr{L}(\boldsymbol{\gamma}; \boldsymbol{g}, \boldsymbol{A}, \boldsymbol{b}) + \lambda \|\boldsymbol{\gamma}\|_{1,2} \tag{3.19}$$

It turns out that for a given $\{\boldsymbol{g}, \boldsymbol{A}, \boldsymbol{b}\}$ one can obtain an analytical expression for $\lambda^\star$ so that the solution to above problem is $\boldsymbol{\gamma} = \mathbf{0}$ [7]. Therefore, $\lambda^\star$ serves as an initial value for computing the optimal λ. Further, a unique aspect of single-molecule localization microscopy concerns its simple signal model consisting of images of blinking molecules. This simplicity allows to obtain an iterative strategy to compute an optimal value for λ. For 2D imaging we first focus on tuning λ for recovering an isolated molecule. Note that in principle we should specify a reasonable signal-to-background (SBR) level so that $\lambda^\star$ can be calculated. Our results show that SBR= 1 exhibits robustness against false positives (FP) and false negatives (FN) (see Fig. S2). The iterative strategy essentially involves decreasing λ starting from $\lambda^\star$ and computing mean FP and FN rates until a satisfactory performance is reached. To further ensure robustness against PSF overlap, we generate images of two overlapping molecules and further tune λ around the optimal λ for the isolated case. This simple tuning strategy ensures robustness against both SBR and various PSF overlaps with a minimal bias at a separation distance of 0.82 FWHM of the Gaussian PSF (see Fig. S4).

We extend the above iterative strategy for 3D imaging with diverse PSFs. To be concrete, we consider K sampled z planes. We can compute $\lambda^\star$ for each plane by restricting the optimization over individual axial planes to obtain $\{\lambda_1^\star, \ldots, \lambda_K^\star\}$. Similar to 2D imaging, we consider isolated image of molecules at various z planes at SBR= 1 to tune λ with respect to mean FP and FN rates. To ensure robustness against PSF overlaps we further tune λ by adding a constant term to it. This strategy exhibits robustness against various SBRs and 3D PSF overlaps for the tetrapod PSF (see Fig. S6).

4. DESCRIPTION OF IMAGE FORMATION MODEL

We consider a uniform labeling density of 0.04 molecules/nm^2 on the surface of the cylinders representing microtubulues. A continuous temporal emission model accounts for stochastic nature of molecular emission (see section 4. A). The labeling density together with the temporal emission characteristics, e.g., on rate and mean switching time, impose a mean blinking density, which represents the average number of active molecules per the area occupied by the sample. For each molecule, we generated photon counts per burst according to a Poisson distribution whose mean is proportional to a chosen emission brightness. We model photon emission without photobleaching in order to directly test algorithmic performance and avoid image artifacts from insufficient sampling of the biological structure [8]. Additionally, the number of frames is selected such that each molecule blinks on average once, which ensures an asymptotic statistical performance analysis.

A. Temporal emission model for a molecule

Motivated by experimental measurements, we model the temporal emission of a SM as a continuous-time Markov process $\mathscr{E}_t$. We define two states for the process, *bright* and *dark* states, representing a molecule that emits fluorescence in response to an excitation laser, and a molecule that does not respond to an excitation laser, respectively. We can compactly describe the process by successive states visited by $\mathscr{E}_t$, $\{X_0, X_1, X_2, \cdots\}$, and the time of each transition $\{T_0, T_1, T_2, \cdots\}$. Interestingly, it turns out that $\{X_n, n \in \mathbb{N}\}$ forms a Markov chain and that $L_n = T_{n+1} - T_n$, called the lifetime of X_n, follows an exponential distribution with parameters depending on X_n [9] (Fig. S1). We assume an exponential distribution for number of photons emitted during the bright state. In general, a realized lifetime of the bright state may extend more than a single frame. In this case, for each frame that is entirely or partially covered by the realized lifetime, an exponential random variable is obtained with a mean equal to the time the corresponding frame contributes to the realized lifetime multiplied by the emission rate. The emission "intensity" represents the mean emission rate multiplied by mean lifetime. Consequently, the number of photons for each frame is drawn from the corresponding exponential distribution.

A.1. Relation between mean molecular blinking density and transition rates

Let's denote the life-time of bright and dark states by l_b and l_d, respectively. We define the transition rate from bright state to dark state (dark state to bright state) by $k_\mathrm{off} = 1/l_\mathrm{b}$ ($k_\mathrm{on} = 1/l_\mathrm{d}$). It turns out that the mean switching cycle rate of a SM is given by $\frac{k_\mathrm{on}k_\mathrm{off}}{(k_\mathrm{on}+k_\mathrm{off})}$ [9]. Therefore, the mean number of molecular blinking events per each frame is given by:

$$\frac{k_\mathrm{on}k_\mathrm{off}}{(k_\mathrm{on}+k_\mathrm{off})} \cdot T_\mathrm{f} \cdot \mathrm{TotNum}, \tag{4.1}$$

where T_f is the camera exposure time and TotNum is the total number of molecules labeling the structure. Finally, the mean molecular blinking density represents the mean number of molecular blinking events per frame divided by the rectangular area that the sample structure occupies in the object space.

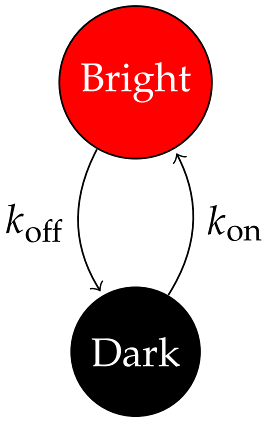

Fig. S1. Two states Markov chain with exponential transitions. Each state is characterized by a parameter $k = \frac{1}{l}$, where l is the mean life-time of that state and that k is the mean rate at which the transition to the other state occurs. Here, k_off denotes the transition rate from the bright state to the dark state, whereas k_on represents the transition rate from the dark state to the bright state. The mean switching rate is given by : $\frac{k_\mathrm{on}k_\mathrm{off}}{(k_\mathrm{on}+k_\mathrm{off})}$.

B. Simulated images and PSF model

A 3D Gaussian PSF was used [10] to model the imaging system for generating 2D SMLM datasets. We elected following parameters: (wavelenght) $\Lambda = 550$ nm; (numerical aperture) NA $= 1.4$; (refractive index of immersion oil) $n = 1.53$; and (defocus) $z_d = 400$ nm. For each molecule with axial position z, the standard deviation of the corresponding Gaussian PSF was set to $\sigma_z = \frac{0.21\Lambda}{\mathrm{NA}\exp(\log(2)|z/z_d|)}$. To obtain 3D PSFs, we used a scalar propagation field derived from the Fresnel approximation [11]. The pixel size of the CCD camera was set to 58.5 nm. The images were obtained by computing the PSF at a high-resolution grid of size 5.85 nm and integrating over a pixel size of 58.5 nm. A uniform background of 40 photons/pixel was added to each pixel prior to applying Poisson distributed photon shot noise.

5. PERFORMANCE METRICS

A. Recall, precision, Jacard, and root-mean-square error

Given a set of localizations and corresponding ground truths for each frame, we first match each localization to a specific ground truth using Hungarian matching algorithm [12]. Specifically, a matching is acceptable if the distance between the localized molecule and its matched ground truth is smaller than 6 σ_CRLB. The detection performance metrics, i.e., the recall, precision, and Jacard as well as root-mean-square error (RMSE) are computed based on the matched pairs. Let TP, FP, and FN denote the number of true positives, false positives, and false negatives, respectively. We then have [10]

$$\mathrm{recall} = \frac{\mathrm{TP}}{\mathrm{TP}+\mathrm{FN}}, \quad \mathrm{precision} = \frac{\mathrm{TP}}{\mathrm{TP}+\mathrm{FP}}, \quad \mathrm{Jacard} = \frac{\mathrm{TP}}{\mathrm{TP}+\mathrm{FN}+\mathrm{FP}}. \tag{5.1}$$

The root-mean-square error (RMSE) is calculated across all the matched pairs as follows [10]:

$$\mathrm{RMSE} = \sqrt{\frac{1}{n}\sum_{i=1}^{n}\left((x_i - \hat{x}_i)^2 + (y_i - \hat{y}_i)^2\right)}, \tag{5.2}$$

where (x_i, y_i) and $(\hat{x}_i, \hat{y}_i)$ represent 2D coordinates of the i_th ground-truth point and its matched estimate, respectively.

B. Clustering

To obtain clusters from a SMLM dataset we set the minimum number of localizations per cluster to 5 [13]. Further, to account for localization precision (σ_{CRLB}) and vesicle size (r_{v}) we set the search radius to $r_{\text{v}} + \sigma_{\text{CRLB}}$. Once clusters are inferred, cluster size is computed using principal-component analysis (PCA). The axes' lengths of the clusters correspond to the square root of the principal components (eigenvalues) multiplied by 2.14, equivalent to a 97% confidence ellipse.

6. RECOVERY ALGORITHMS

A. FALCON

Reconstruction using FALCON [14] was performed on MATLAB with `FALCON-CPU-rel2` and default parameters (sparsity para= 2.5 and normal speed option) unless otherwise specified. The effectiveness of these parameters were corroborated by analyzing isolated images of molecules as well as images of two closely-spaced molecules in simulations.

B. Super-resolution radial fluctuations (SRRF)

Analyses by SRRF [6] were performed using the corresponding ImageJ plug-in. The parameters for SRRF-TRPPM, SRRF-TRAC2, SRRF-TRAC3, and SRRF-TRAC4 were selected as default values, except for the magnification factor. The dataset presented in Fig. S9,S10 were analyzed with a magnification factor of 3, for the datasets in Fig. S11,S12 a magnification factor of 6 was used, and for the experimental datasets in Fig. S15,S16 magnification factor of 4 was used.

Table S1 provides detailed comparison of FALCON, SRRF, and RoSE-C in terms of number of localizations and running time.

C. ThunderSTORM

ThunderSTORM's ImageJ plug-in [15] was used to perform low-density reconstructions using the integrated Gaussian PSF and maximum likelihood estimation with a fitting radius of 3 pixels.

7. QUANTIFYING STRUCTURAL BIAS IN MEASURING THE SEPARATION AND APPARENT LABELING DENSITY OF VESICLES USING SRRF AND ROSE-C

In this section, we quantify the separation and relative density of recovered vesicles (i.e., the number of blinking events or brightness of each vesicle) from simulated SMLM datasets. A low-density (LD) dataset containing 4 vesicles uniformly-labeled with blinking molecules (Fig. S9(a)) was analyzed by ThunderSTORM (Fig. S9(b)), while RoSE-C (Fig. S9(c)) and SRRF (Fig. S9(d)) analyzed a HD dataset with the same total number of blinking events. To measure the separation between two vesicles, the localizations within the region of interest (see box in Fig. S9(b)) were projected onto the x axis and normalized relative to the total number of localizations.

The projected profiles of the two vesicles (Fig. S9(e)) remarkably show the robustness of RoSE-C in accurately resolving the distance between vesicles (-5 nm bias or -3%) and their relative densities with a normalized visibility of 1 compared to LD imaging (separation bias $= 0.5$ nm (0.3%) and normalized visibility = 1). SRRF-TRPPM, which applies pixel-wise autocorrelation on radiality maps, has a poor visibility of 0.64. Importantly, it exhibits a bias of -20 nm (-13%) in measuring the separation distance. SRRF-TRAC4, which applies a fourth-order autocumulant, increases the normalized visibility to 1. However, it incurs a bias of $+40$ nm (27%) in measuring the separation between vesicles and exhibits a significant bias in estimated labeling density. Interestingly, the arrangement of vesicles affects the accuracy of each SRRF algorithm differently due to non-zero cross-correlation terms in the cumulant analysis [6] (Fig. S10). This analysis reveals that the apparent visibility between structures, which is unity for SRRF-TRAC2, TRAC3, and TRAC4, ignores the structural biases present in both estimated position and labeling density.

Because the accuracy of cumulant analysis and pixel-wise-correlation improves with an increasing number of uncorrelated frames [16], we have performed simulations to separate the structural errors from those that are due to an insufficient number of blinking events. Surprisingly, even when each activated molecule blinks, on average, 4.8 times over 480 frames, the central vesicles appear to shift $+20$ nm toward the right-most vesicle for SRRF-TRAC4 (Fig. S11). This shift is minimal for both LD imaging (-8 nm) and RoSE-C (-9 nm). Additionally, SRRF-TRAC4 overestimates the average labeling density of the two left-most vesicles by 20%, while it underestimates the labeling density of the right-most ones by 18% (Fig. S12). These observations show that localization errors and biases in measuring labeling density affect one another.

8. ROLE OF PSF DEGENERACY IN CAUSING STRUCTURAL BIASES

To gather insight into the structured mislocalizations in the recovered NPCs using the tetrapod PSF, we consider overlapping images of molecules located within a small volume $[-60,\ 290] \times [14,\ 219] \times [70,\ 470]$ nm^3. The image (Fig. S19) generated by a set of molecules with various brightnesses and positions can be closely approximated by another set of molecules of distinct brightnesses and positions. This example illustrates a high degree of linear dependency [17] of tetrapod PSF sampled within a relatively small region; that is, multiple arrangements of molecules can generate very similar images on the camera.

This degeneracy fundamentally limits the performance of any sparse recovery algorithm [17]: it is easier to recover the true signal if there is a small degree of linear dependence between the columns of the PSF matrix A. If there is a large degree of linear dependence, then accurate recovery is not guaranteed. However, these theoretical results do not reveal the role of the structure itself influencing the bias (Fig. S18).

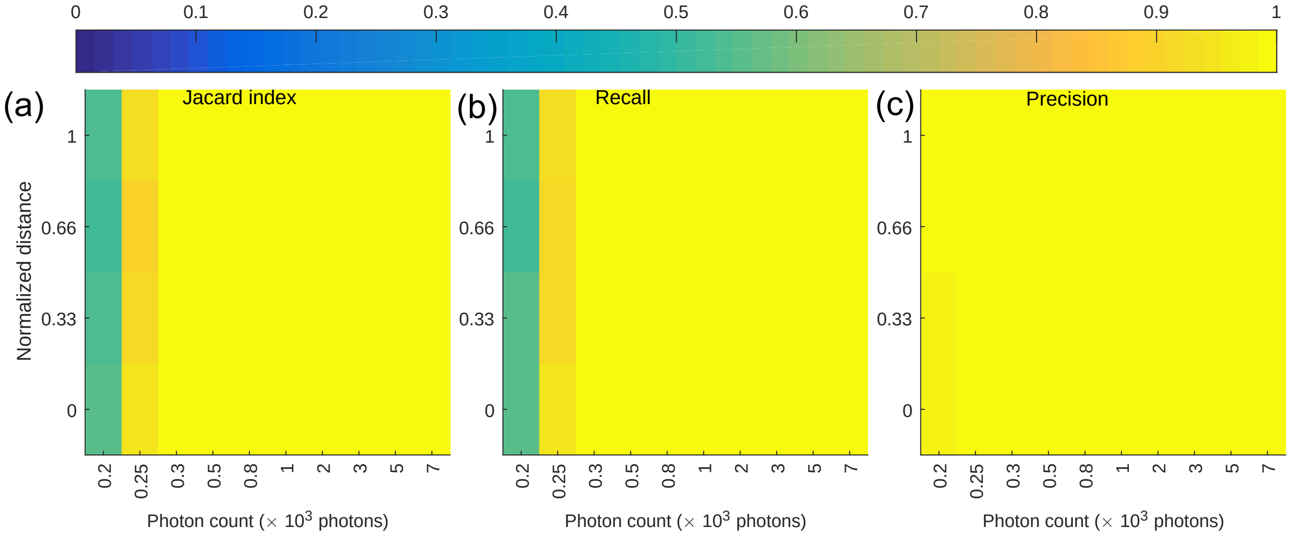

Fig. S2. Detection performance (2D imaging) of RoSE in recovering a molecule located at various distances from the nearest grid point for a range of SBRs. (a) Jacard. (b) Recall. (c) Precision. Normalized distance represents the ratio between the distance of a molecule from a grid point and the distance of that grid point to middle of the grid. The mean background was set to 40 photons/pixel. For each case, 500 independent frames were analyzed.

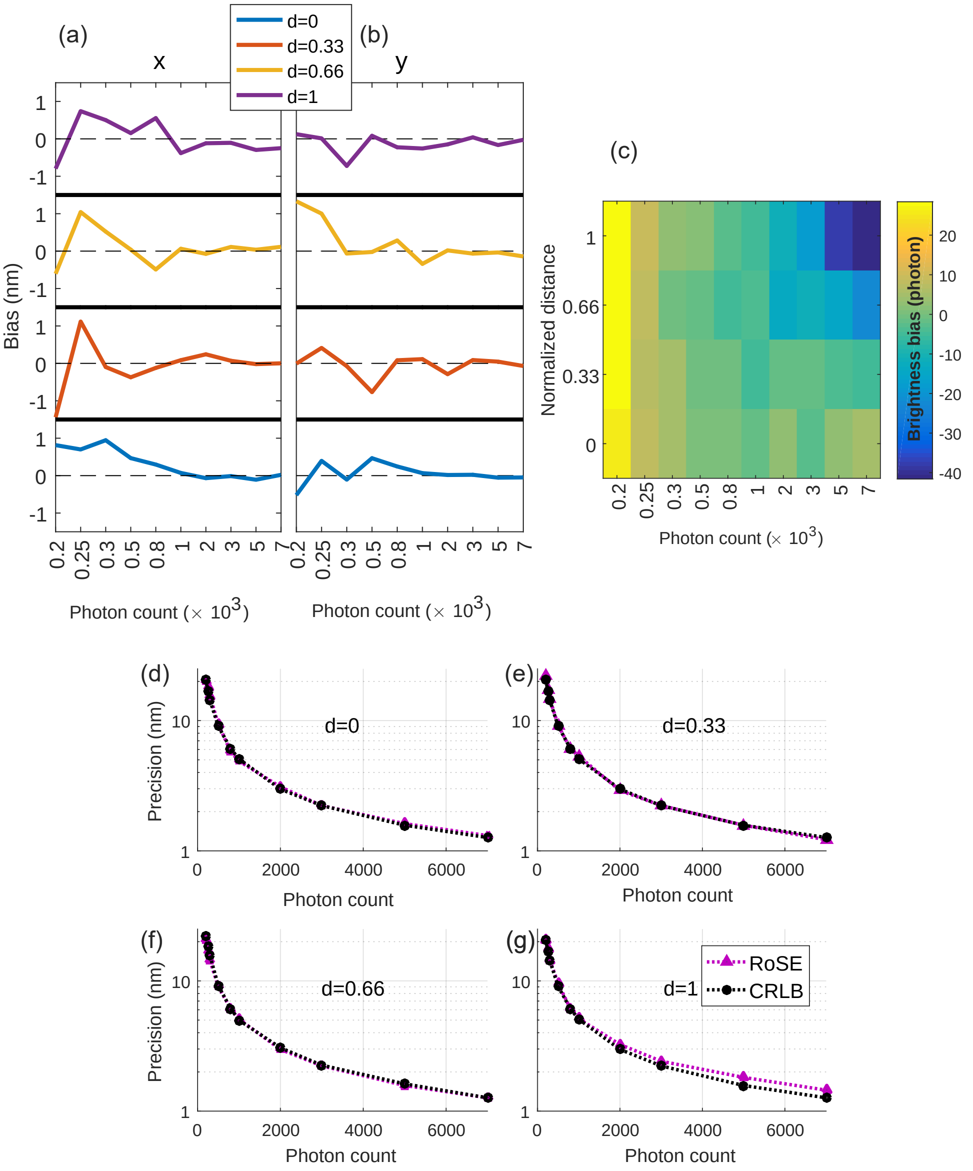

Fig. S3. Bias and precision analysis (2D imaging) of RoSE in localizing a molecule located at various distances from a grid point for a range of SBRs . (a) Bias along x and (b) y in localizing a molecule. (c) Brightness bias in localizing a molecule. Localization precision of RoSE compared to CRB in recovering a molecule for a normalized distance of (d) d= 0, (e) d=0.33, (f) d= 0.66, and (g) d= 1, respectively. The mean background was set to 40 photons/pixel. For each case, 500 independent frames were analyzed.

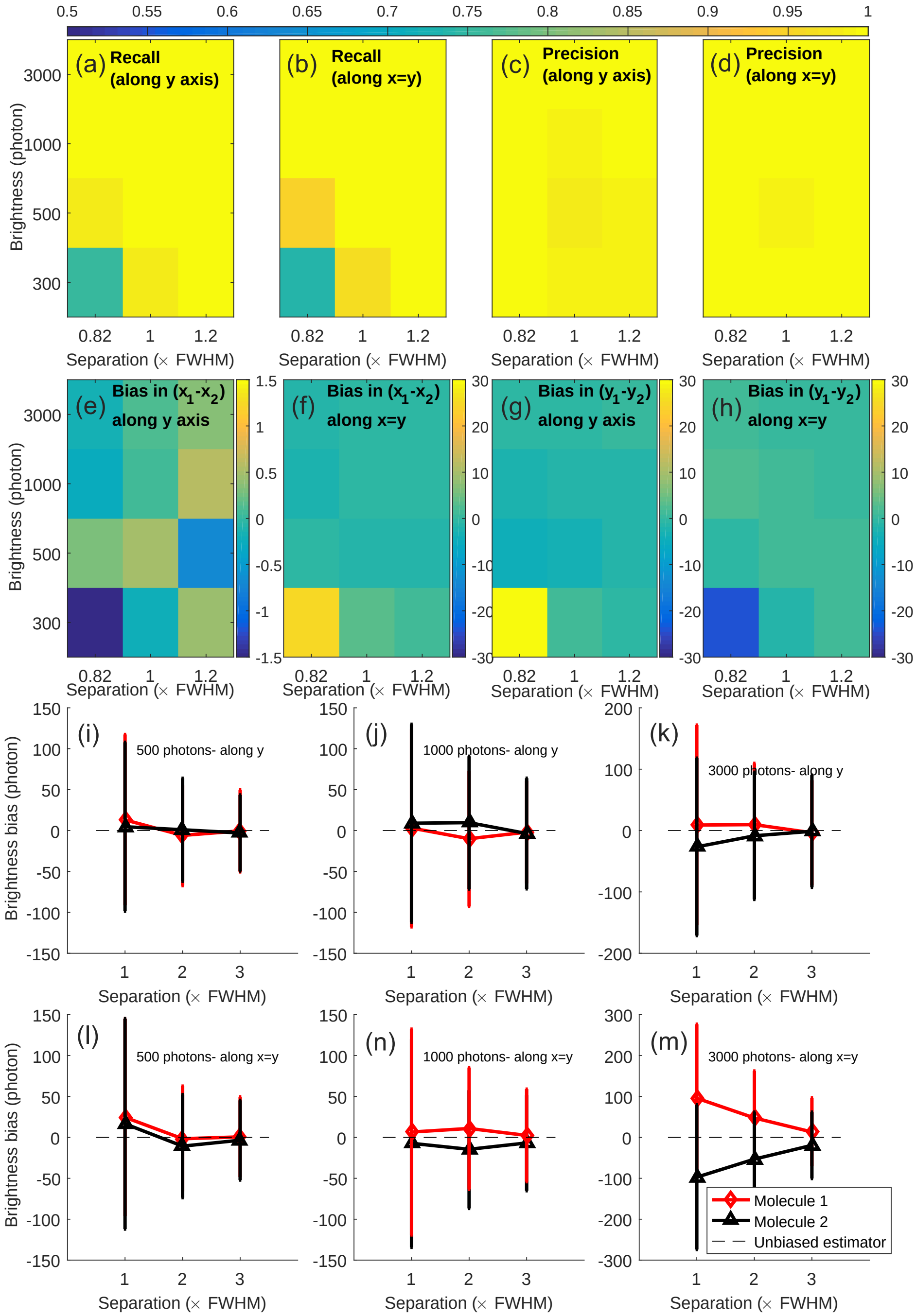

Fig. S4. Detection and bias of RoSE in recovering two closely-spaced molecules as a function of separation distance and SBR. (a) Mean recall in detecting two molecules separated along y axis for various brightnesses. (b) Same as (a) but the two molecules are separated along x=y. (c) Precision in detecting two molecules separated along y axis for various brightnesses. (d) Same as (c) but the two molecules are separated along x=y. (e) Bias in $(x_1 - x_2)$ in localizing two molecules separated along y axis for various brightnesses. (f) Same as (e) but the two molecules are separated along x=y. (g) Bias in $(y_1 - y_2)$ in localizing two molecules separated along y axis for various brightnesses. (h) Same as (g) but the two molecules are separated along x=y. (i) Brightness bias in recovering two molecules separated along y with mean emission intensities of 500 photons. (j) Same as (i) but two molecules have mean emission intensities of 1,000 photons. (k) Same as (i) but two molecules have mean emission intensities of 3,000 photons. (l) Same as (i) but two molecules are separated along x=y. (n) Same as (j) but two molecules are separated along x=y. (m) Same as (k) but two molecules are separated along x=y. In (a-m) a mean background of 40 photons/pixel was used.

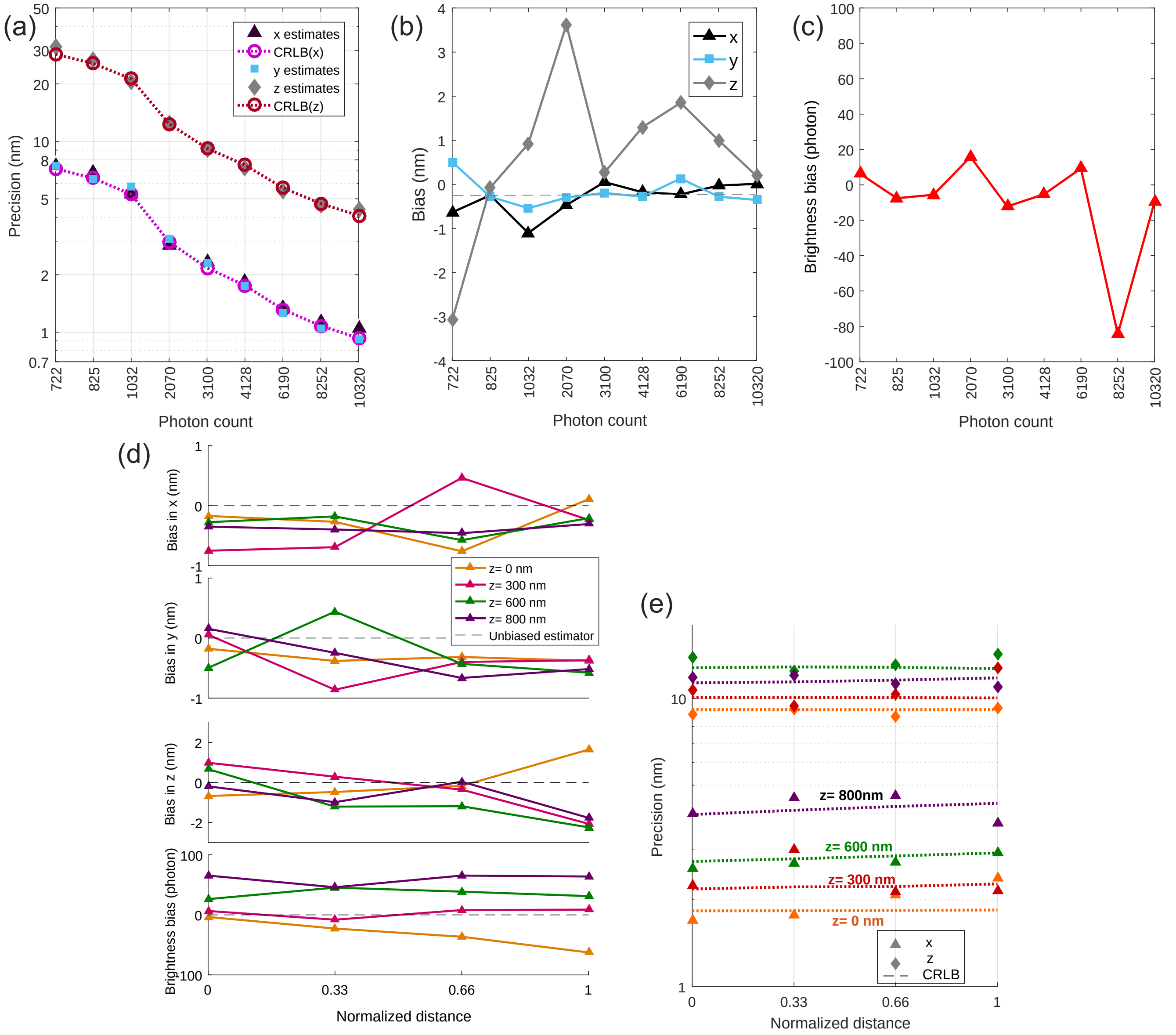

Fig. S5. Bias and localization precision of RoSE in recovering a molecule using the tetrapod PSF. (a) Localization precision of RoSE compared to CRB in recovering a molecule located across a depth range spanning $[-1,1]$ μm. The precision at each photon count was obtained by analyzing 200 measurements. (b) Localization bias of RoSE along x (black triangles), y (cyan squares), and z (grey diamonds) corresponding to measurements in (a). (c) Brightness bias of RoSE corresponding to measurements in (a). (d) Localization bias of RoSE as a function of molecule's distance from the nearest grid point. Molecules were placed at $z = \{0\ (\text{orange}), 300\ (\text{red}), 600\ (\text{green}), 800\ (\text{purple})\}$ nm. (e) Localization precision of RoSE along x (triangles) and z (squares) compared to CRB (dashed curve) as a function of molecule's distance from the nearest grid point. For each case in (a-e), 200 independent frames were analyzed and a mean background of 40 photons/pixel was considered.

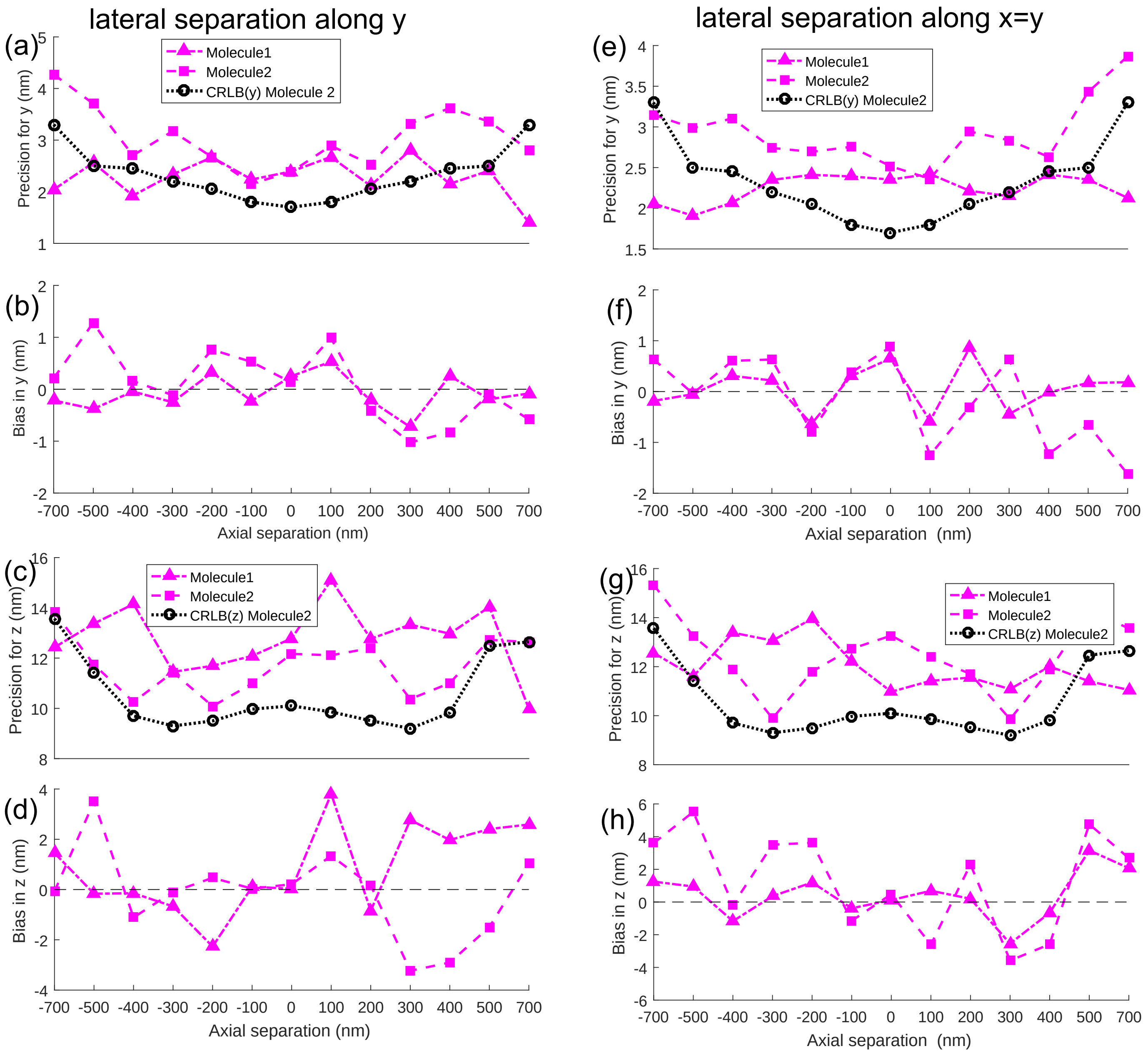

Fig. S6. Lateral and axial localization bias and precision of RoSE in recovering two closely-spaced molecules using the tetrapod PSF. (a) Precision and (b) bias along y in localizing two closely-spaced molecules located at $(x_1, y_1) = (0, 0)$ nm and $(x_2, y_2) = (0, 142)$ nm at an SBR of 3 corresponding to $3,100$ photons detected from each molecule with a mean background of 40 photons/pixel. Triangles (dotted curve) represent the precision and bias of localizing molecule 1, while squares (dashed curve) represent the precision and bias of localizing molecule 2. The ideal limits of localization precision and bias are plotted in black. A positive bias for molecule 1 indicates a bias toward the second molecule, while a negative bias for molecule 2 indicates a bias toward the first molecule. (c) Precision and (d) bias along z in localizing the two closely-spaced molecules in (a,b). (e) Precision and (f) bias along y in localizing two closely-spaced molecules located at $(x_1, y_1) = (0, 0)$ nm and $(x_2, y_2) = (100, 100)$ nm at an SBR of 3 corresponding to $3,100$ photons detected from each molecule with a mean background of 40 photons/pixel. Triangles (dotted curve) represent the precision and bias of localizing molecule 1, while squares (dashed curve) represent the precision and bias of localizing molecule 2. The ideal limits of localization precision and bias are plotted in black. A positive bias for molecule 1 indicates a bias toward the second molecule, while a negative bias for molecule 2 indicates a bias toward the first molecule. (g) Precision and (h) bias along z in localizing the two closely-spaced molecules in (e,f).

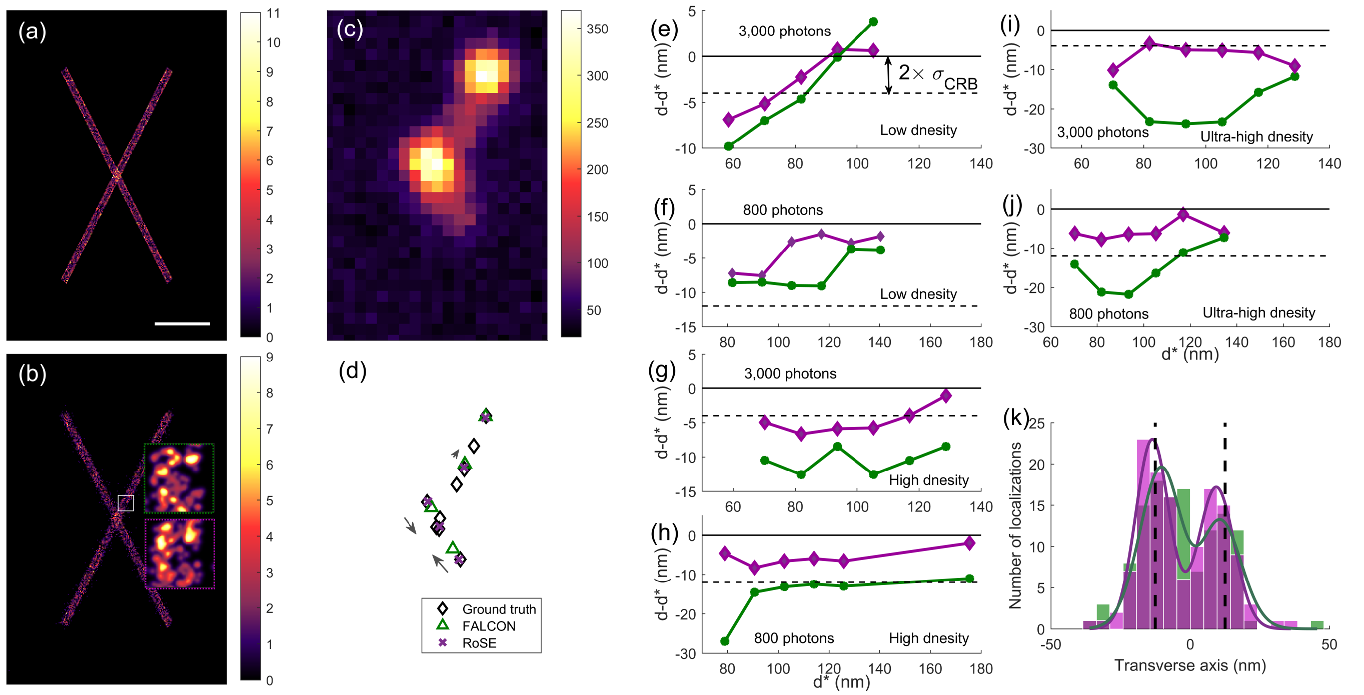

Fig. S7. Structural bias of two crossing microtubules recovered by RoSE (purple) and FALCON (green). The low, high, and ultra-high densities correspond to blinking densities of 4.9×10^{-6}, 1.3×10^{-5}, and 1.9×10^{-5} molecules/nm^2, respectively. (a) Simulated ground-truth structure. Color bar: number of molecules per 5×5 nm^2. (b) Recovered structure by RoSE at low-blinking density and mean emission intensity of 3,000 photons. Color bar: number of localizations per 5×5 nm^2. Inset: Magnified projection of the boxed region onto the axis transverse to microtubule for RoSE (purple) and FALCON (green). The insets were obtained by blurring the localizations with a Gaussian distribution with a 2.3 nm standard deviation. (c) A representative simulated camera frame for high-blinking density and mean emission intensity of 800 photons. Color bar: number of photons per 58.5×58.5 nm^2. (d) Recovered molecules corresponding to (c) using RoSE and FALCON. The arrows show the direction of localization errors for FALCON with corresponding magnitudes. (e,f) Mean separation bias (d-d*) between crossing MTs at various true separations (d*) for low-blinking density, and mean emission intensities of 3,000 and 800 photons, respectively. (g,h) Mean separation bias (d-d*) between crossing MTs at various true separations (d*) for high-blinking density, and mean emission intensities of 3,000 and 800 photons, respectively. (i,j) Mean separation bias (d-d*) between crossing MTs at various true separations (d*) for ultra-high, blinking density, and mean emission intensities of 3,000 and 800 photons, respectively. (k) Projected histogram corresponding to insets in (b). Dashed lines represent the positions of the MTs walls. RoSE resolves the MTs walls with better visibility than FALCON (57% larger) . Scale bar: 300 nm.

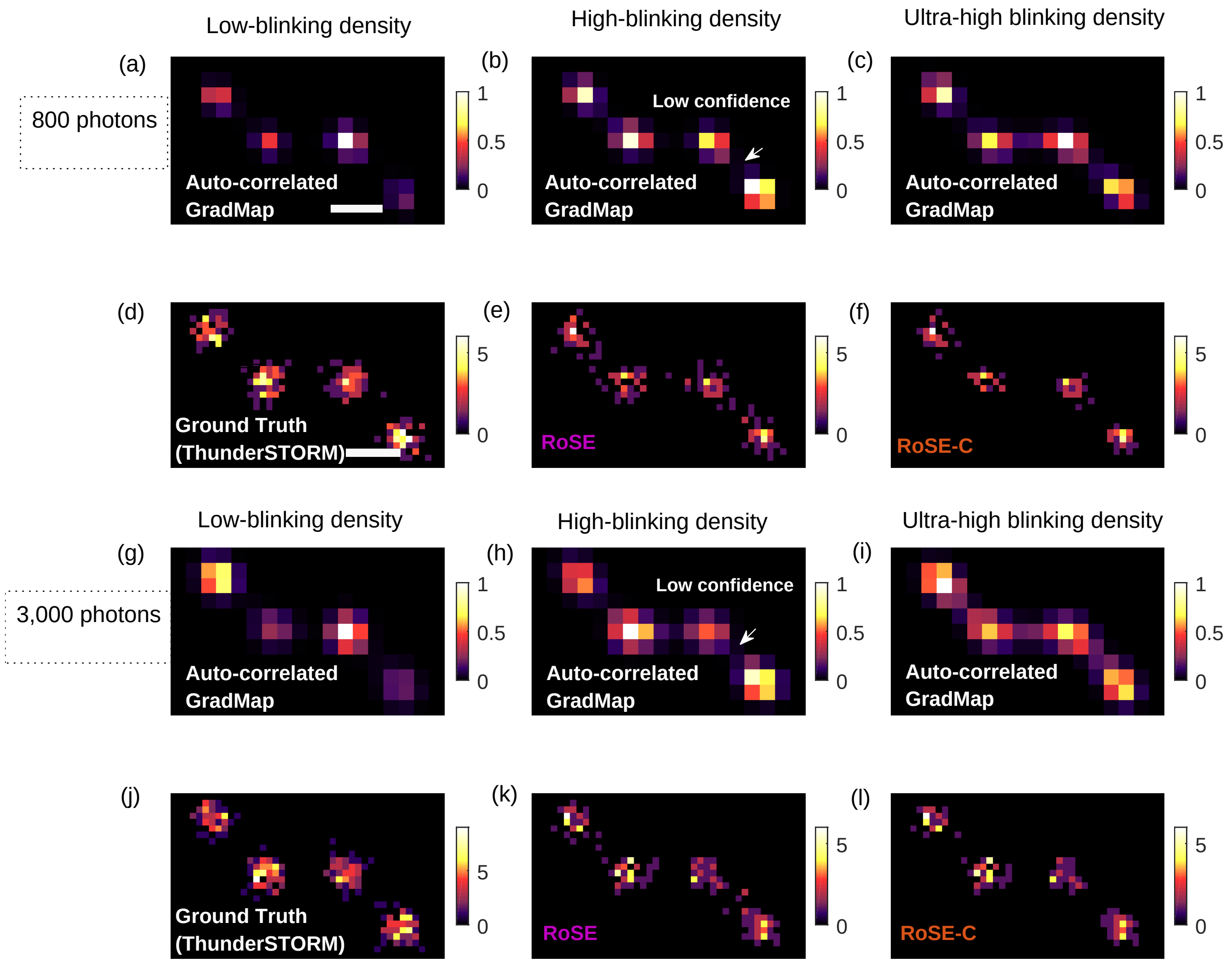

Fig. S8. Eliminating false localizations in recovering simulated packed vesicles using estimated confidence levels. (a-c) Auto-correlated GradMaps for blinking densities of (a) 2.8×10^{-5} (low-blinking density), (b) 7.1×10^{-5} (high-blinking density), and (c) 10×10^{-5} (ultra-high blinking density), respectively. The auto-correlation order was set to 3 (Eq. 3.15). The mean emission intensity was set to 800 photons; the mean uniform background was set to 40 photons per pixel; and a total of 80 frames were used for reconstruction. (d) Ground-truth structure recovered by ThunderSTORM for the high-blinking dataset in (b) using frames with isolated blinking events. (e) Recovered structure by RoSE for the high-blinking dataset in (b). (f) Recovered structure by RoSE-C for the high-blinking dataset in (b). The threshold level in RoSE-C was set to 0.1. (g-l) Same as (a-f), but the mean emission intensity was set to 3,000 photons and the auto-correlation order was set to 2. Scale bars: 100 nm.

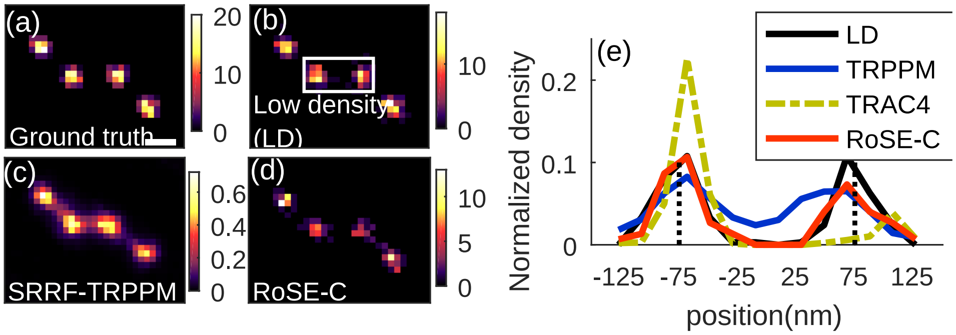

Fig. S9. Bias in measuring vesicle separation and labeling density. (a) Simulated ground-truth structure of four uniformly-labeled vesicles. The mean emission intensity and mean uniform background were set to 3000 photons and 40 photons per pixel, respectively. Scale bar: 100 nm. (b) Structure recovered from low-density (LD) frames using ThunderSTORM. (c) Density map recovered by SRRF-TRPPM. (d) Structure recovered by RoSE-C. Color bars: (a,b,d) number of localizations per 19.5×19.5 nm^2, (c) normalized labeling density. (e) 1D profile of the two middle vesicles (box in (b)) projected onto the x axis for LD, SRRF (TRPPM and TRAC4), and RoSE-C. Dotted lines denote the true centers of the two vesicles.

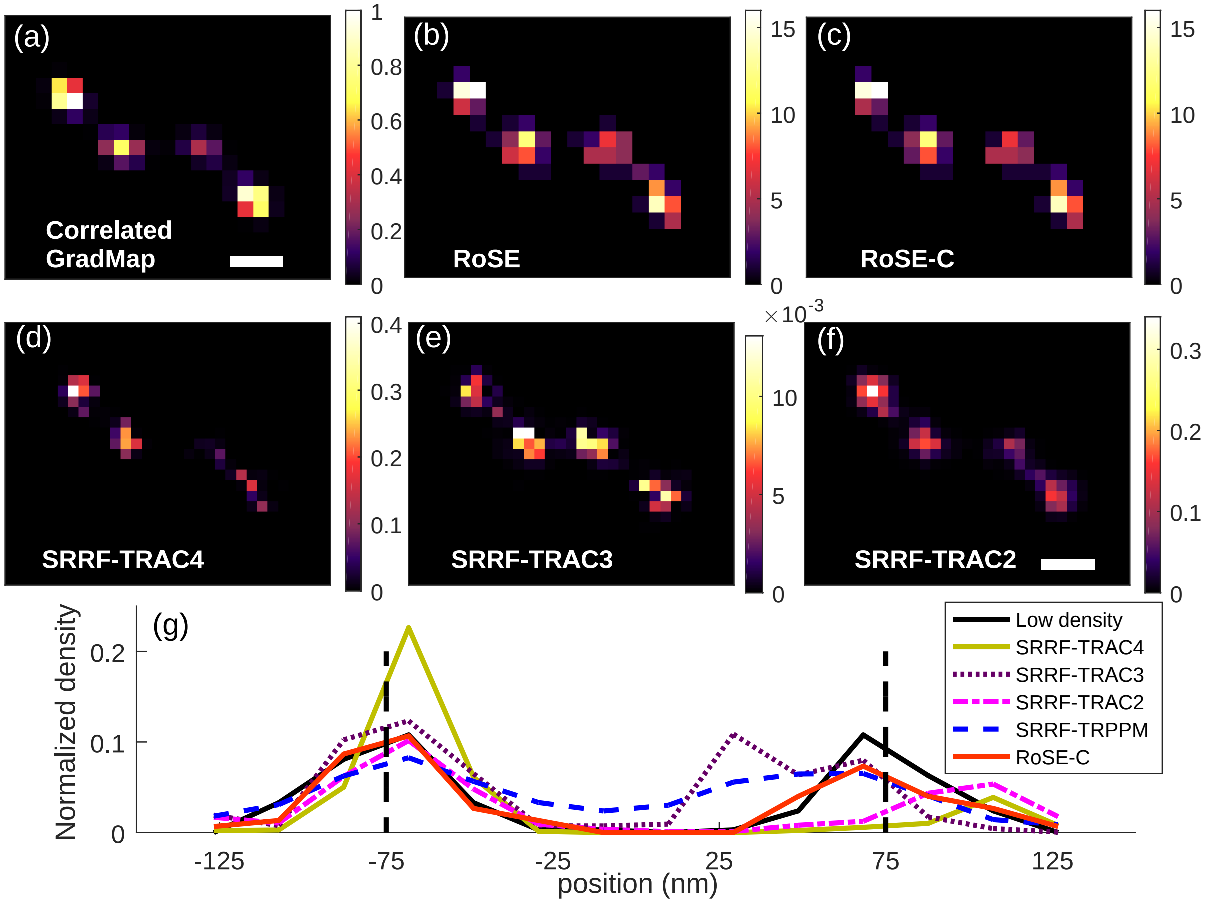

Fig. S10. Bias in measuring vesicle separation. (a) Correlated GradMap with pixel size of 29 nm representing 2 times upsampling of the camera pixel. The GradMap was obtained via temporal autocorrelation of order 2 and a thresholding value of 0.1. (b) Localization histograms of RoSE and (c) RoSE-C. (d) Density map obtained using SRRF-TRAC, (e) SRRF-TRAC3, and (f) SRRF-TRAC2. Color bars: (a) normalized blinking density per 29×29 nm^2, (b,c) number of localizations per 29×29 nm^2, (d-f) normalized blinking density per 19.5×19.5 nm^2. Scale bar: 100 nm. In (d-f) the magnification factor of SRRF was set to 3. (g) 1D profile of the two middle vesicles (box in (f)) projected onto the x axis for low density, SRRF (TRAC4, TRAC3, TRAC2, TRPPM), and RoSE-C. Dotted lines denote the true centers of the two vesicles.

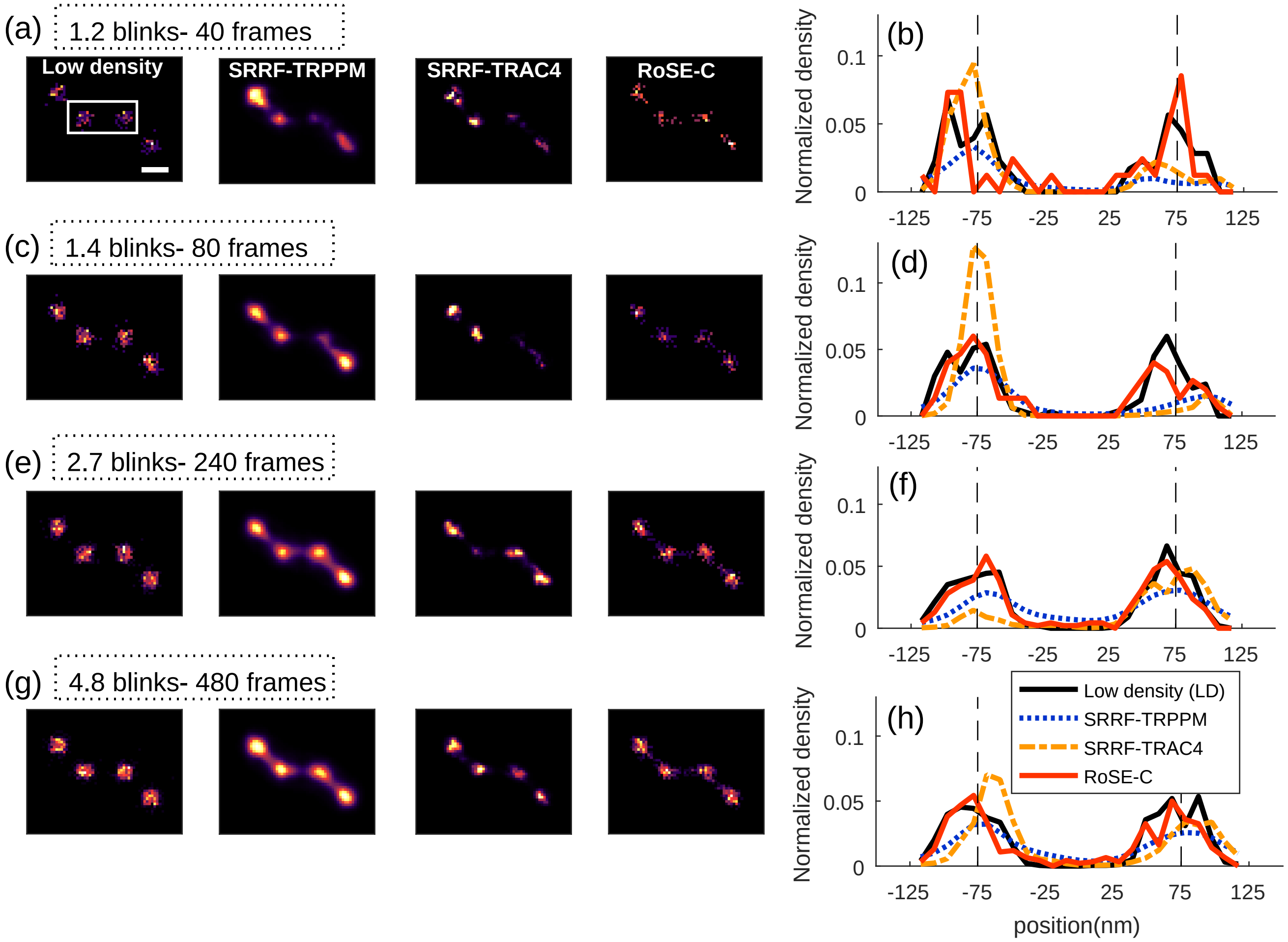

Fig. S11. Effect of number of frames (or mean number of blinks per activated molecule) on errors in measuring vesicle separation. (a) Recovered maps by low density, SRRF (TRPPM and TRAC4), and RoSE-C for a mean number of 1.2 blinks per activated molecules over 40 simulated frames. Scale bar: 100 nm. (b) 1D profile of the two middle vesicles (box in (a)) projected onto the x axis for low density (LD), SRRF (TRPPM and TRAC4), and RoSE-C. Dotted lines denote the true centers of the two vesicles. (c,d) Same as (a,b) but for a mean number of 1.4 blinks per activated molecules over 80 simulated frames. (e,f) Same as (a,b) but for a mean number of 2.7 blinks per activated molecules over 240 simulated frames. (g,h) Same as (a,b) but for a mean number of 4.8 blinks per activated molecules over 480 simulated frames. In (a-h) the magnification factor of SRRF was set to 6 for better accuracy; the mean molecular labeling density, mean blinking density, mean emission intensity, and mean background were set to 0.035 molecules/nm^2, 4.7×10^{-5} molecules/nm^2, 3,000 photons, and 40 photons/pixel, respectively.

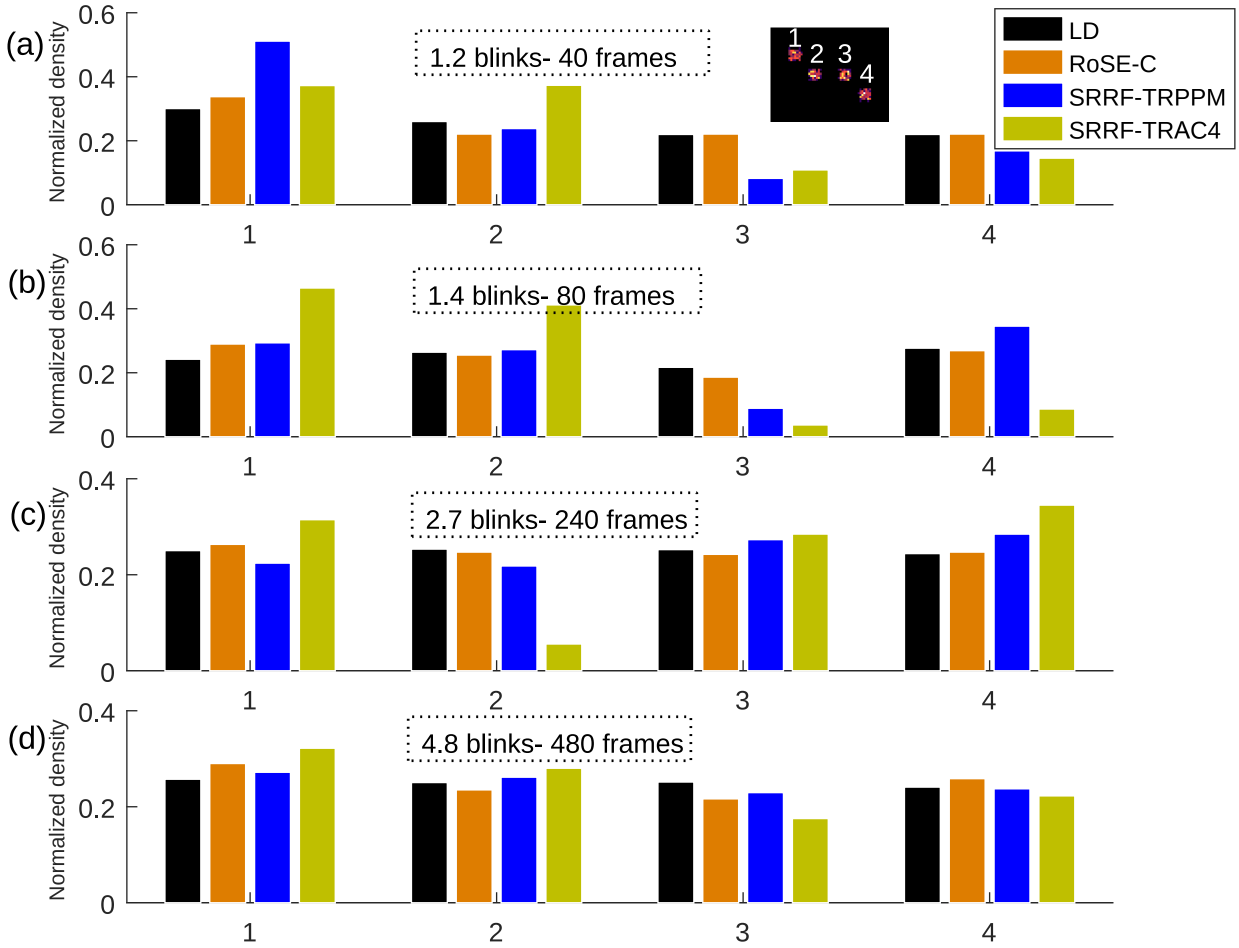

Fig. S12. Effect of number of frames (or mean number of blinks per activated molecule) on errors in measuring normalized labeling density of vesicles. (a) Normalized labeling density of vesicles numbered 1 to 4 recovered by low density (LD), RoSE-C, and SRRF (TRPPM and TRAC4) for a mean number of 1.2 blinks per activated molecules over 40 frames. For computing normalized labeling densities, a box centered at each vesicle was fixed and pixels within that box were integrated. (b) Same as (a) but for a mean number of 1.4 blinks per activated molecules over 80 frames. (c) Same as (a) but for a mean number of 2.7 blinks per activated molecules over 240 frames. (d) Same as (a) but for a mean number of 4.8 blinks per activated molecules over 480 frames. In (a-d) the magnification factor of SRRF was set to 6 for better accuracy; the mean molecular labeling density, mean blinking density, mean emission intensity, and mean background were set to 0.035 molecules/nm^2, 4.7×10^{-5} molecules/nm^2, 3,000 photons, and 40 photons/pixel, respectively.

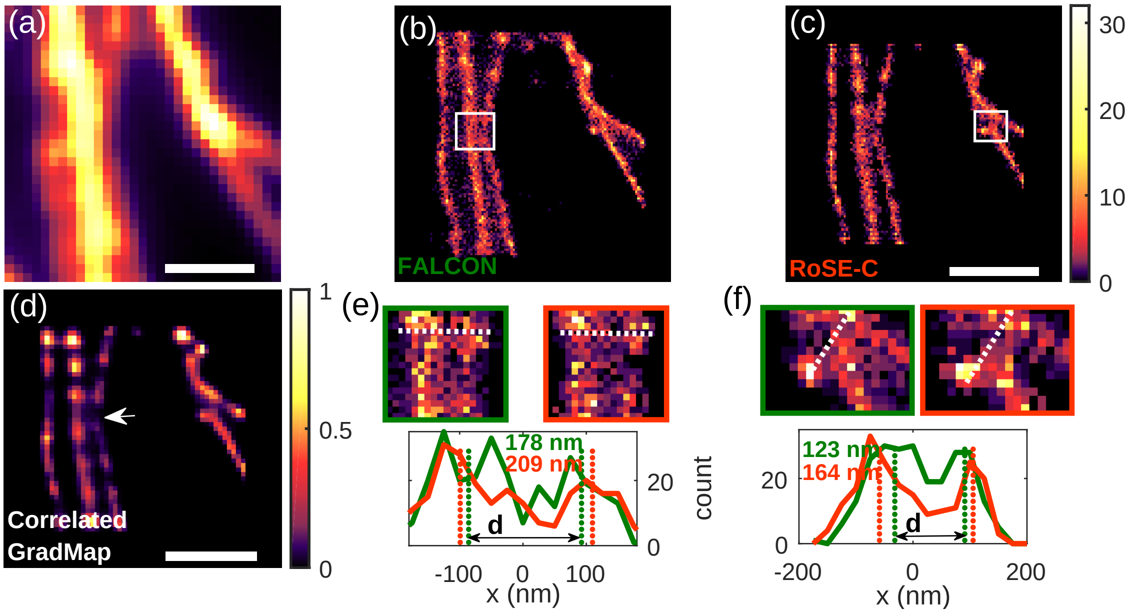

Fig. S13. Structural bias of microtubules from dense, experimental SMLM images using FALCON and RoSE-C. (a) Diffraction-limited sum of the SMLM image stack. (b,c) Histograms of localizations obtained using (b) FALCON and (c) RoSE-C. Color bar: number of localizations per 25×25 nm^2. (d) Correlated GradMap obtained by RoSE-C. White arrow denotes a region containing localizations with low confidence. Color bar: confidence level. (e) Magnified view of the boxed region in (b) for both FALCON (green) and RoSE-C (orange) and projected line plots along the corresponding dotted white lines. (f) Similar to (e), but for the boxed region in (c). The green and orange dotted lines in (e) and (f) denote the double Gaussian fit centers for FALCON and RoSE-C, respectively. The noted separation distance d between the structures is calculated from the double Gaussian fits. The pixel size in (e) and (f) is 25×25 nm^2. Scale bars: 1 μm.

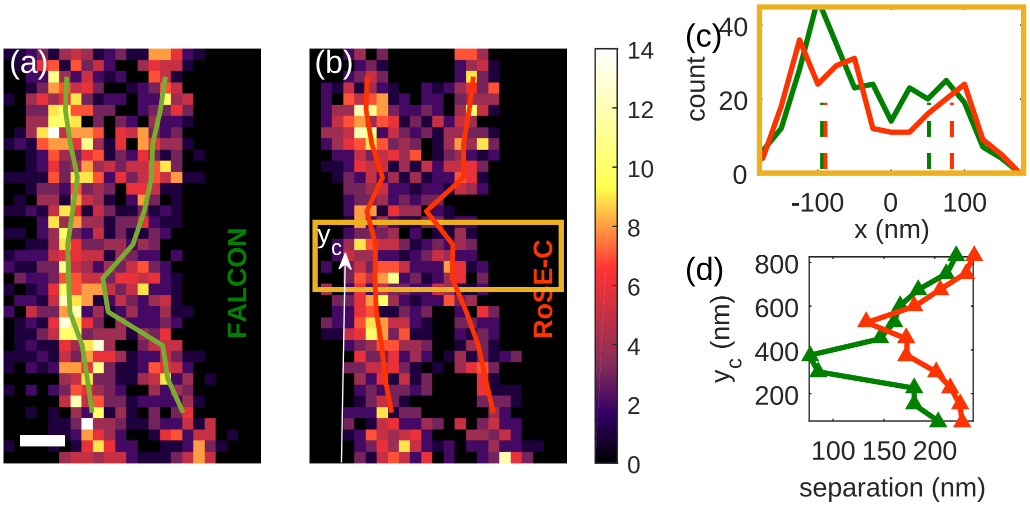

Fig. S14. Structural bias in measuring the separation of microtubules from experimental SMLM images using FALCON and RoSE-C. (a,b) Histograms of localizations obtained using (a) FALCON and (b) RoSE-C. Color bar: number of localizations per 25 × 25 nm^2. The yellow box indicates a sliding window centered at y_c of height 120 nm used to obtain double Gaussian fits. The green and orange curves represent the the measured microtubule structure computed from the centers of double Gaussian fits. (c) Projection of localizations within the orange box in (b) onto x axis for FALCON (green) and RoSE-C (orange). The green and orange dotted lines in (c) denote the double Gaussian fit centers for FALCON and RoSE-C, respectively. (d) Distance separating the two microtubules, as measured by FALCON (green) and RoSE-C (orange) from the green and orange curves in (a,b). Scale bar: 100 nm.

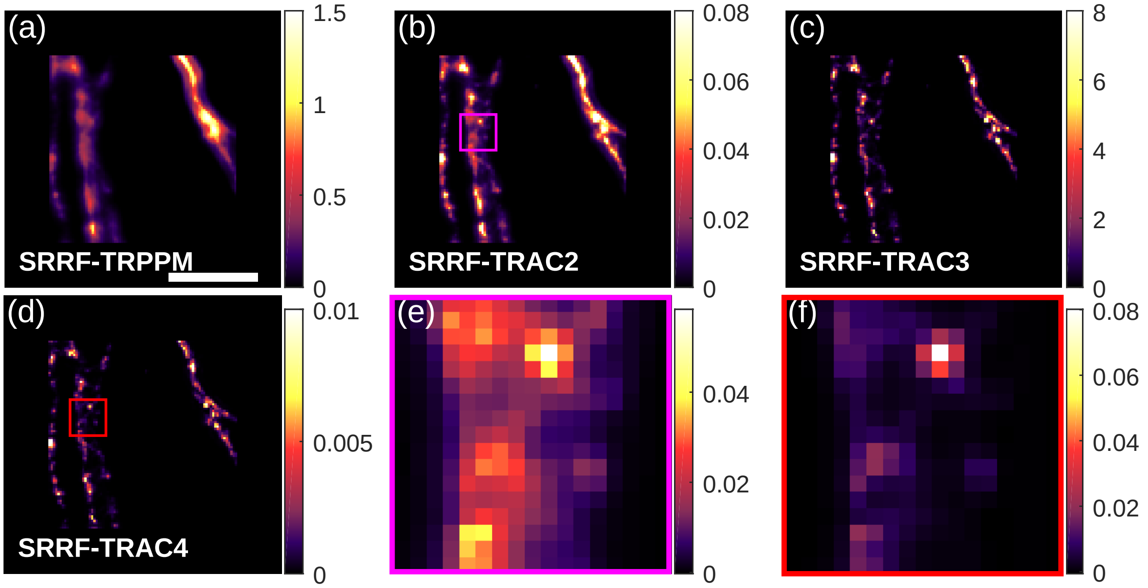

Fig. S15. Images of microtubules recovered from HD experimental blinking data using SRRF algorithms (same data as Fig. S13). Recovered images using (a) SRRF-TRPPM, (b) SRRF-TRAC2, (c) SRRF-TRAC3, and (d) SRRF-TRAC4. (e,f) Magnified views of the boxed region in (b) and (d) for SRRF-TRAC2 (magenta) and SRRF-TRAC4 (red). Scale bar: 1 μm.

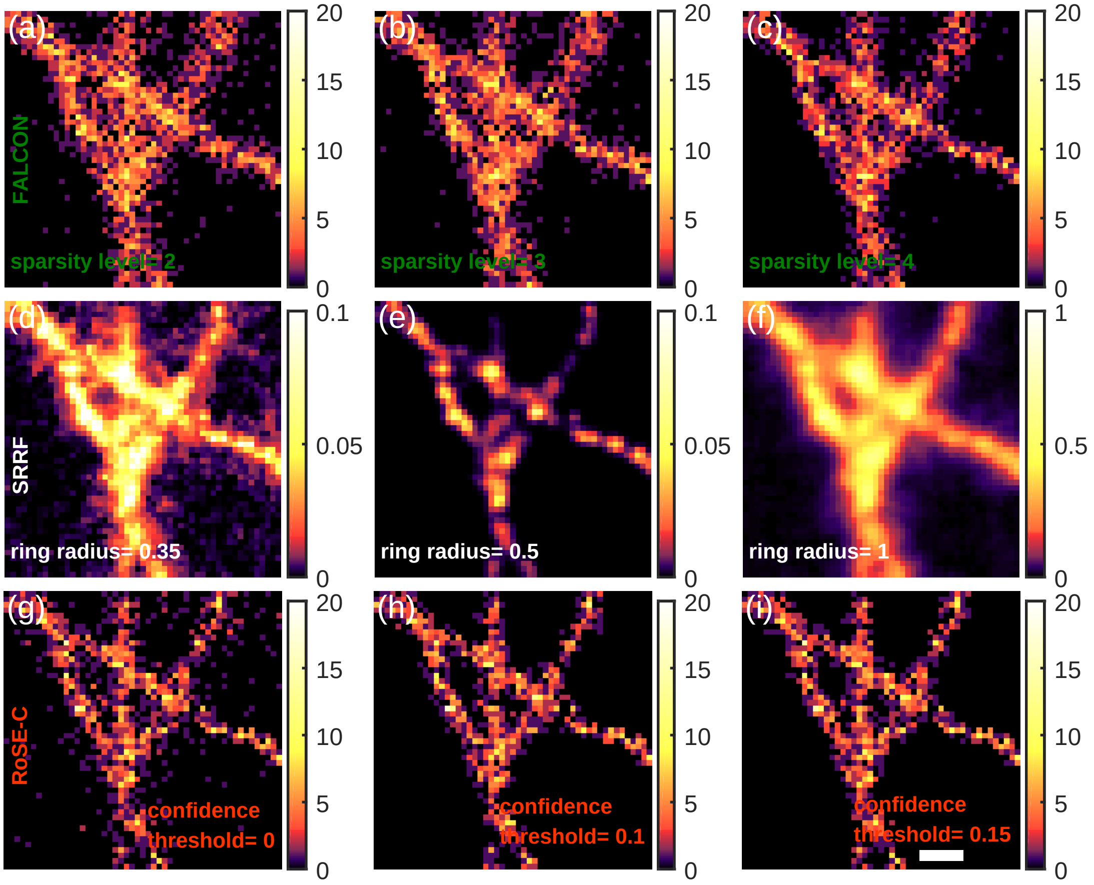

Fig. S16. Images of a dense network of microtubules recovered from HD experimental blinking data using FALCON, SRRF and RoSE-C with various parameters (same data as Fig. 4). Recovered structures using FALCON with a sparsity level of (a) 2, (b) 3, and (c) 4. Recovered structures using SRRF-TRAC2 with a ring radius of (d) 0.35, (e) 0.5, and (f) 1. Recovered structures using RoSE-C with a confidence threshold of (g) 0, (h) 0.1, and (i) 0.15. Scale bar: 100 nm.

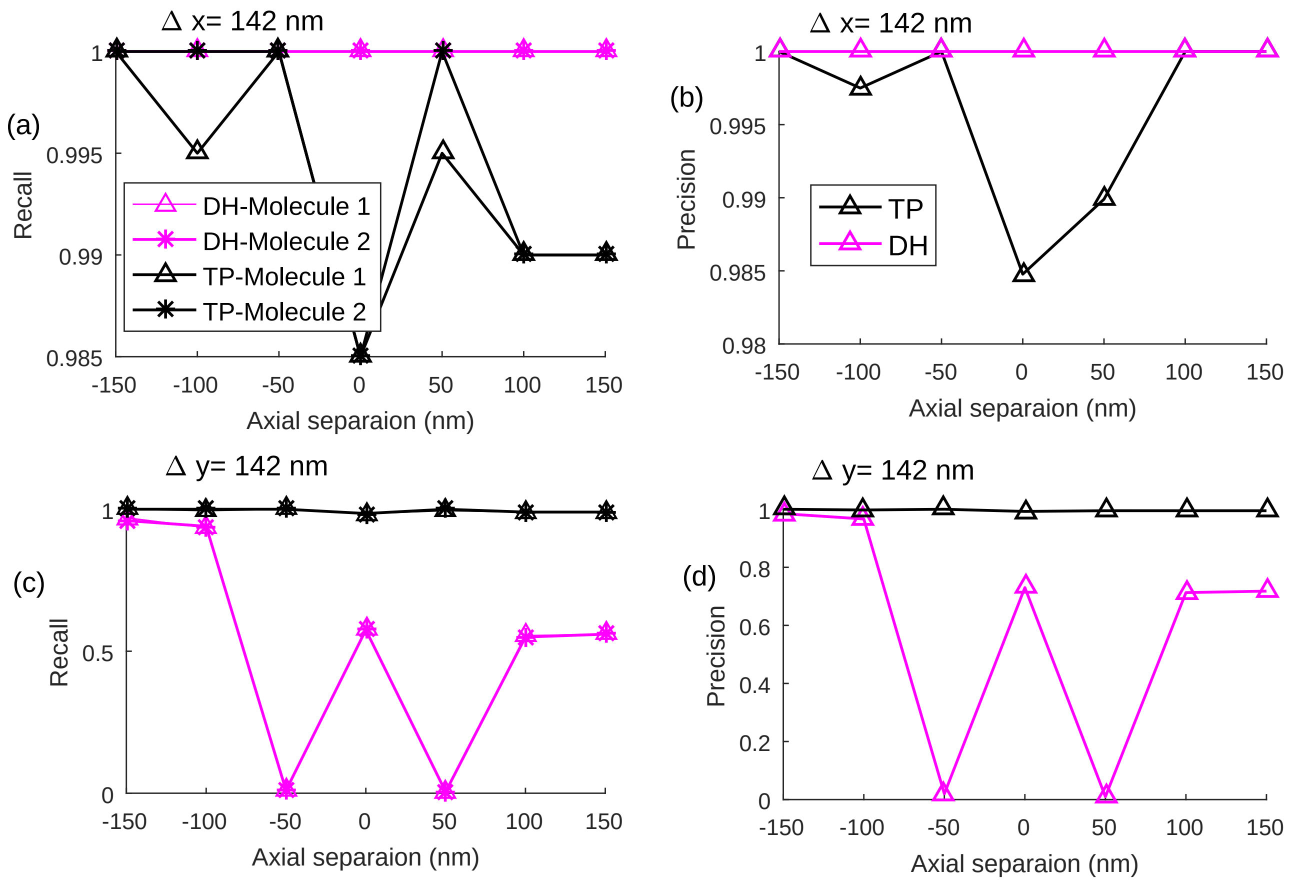

Fig. S17. Detection performance of RoSE in localizing two closely-spaced molecules using the double-helix (DH) and tetrapod (TP) PSFs. (a) Recall and (b) precision in localizing two molecules located at $(x_1, y_1) = (0, 0)$ and $(x_2, y_2) = (142, 0)$ nm, receptively. A mean emission intensity of 3100 photons for both molecules and a mean background of 40 photons/pixel were used. (c,d) Same as (a,b) but for molecules located at $(x_1, y_1) = (0, 0)$ and $(x_2, y_2) = (0, 142)$ nm, receptively. The tetrapod PSF demonstrates a superior performance compared to DH-PSF in resolving two closely-spaced molecules along the y axis.

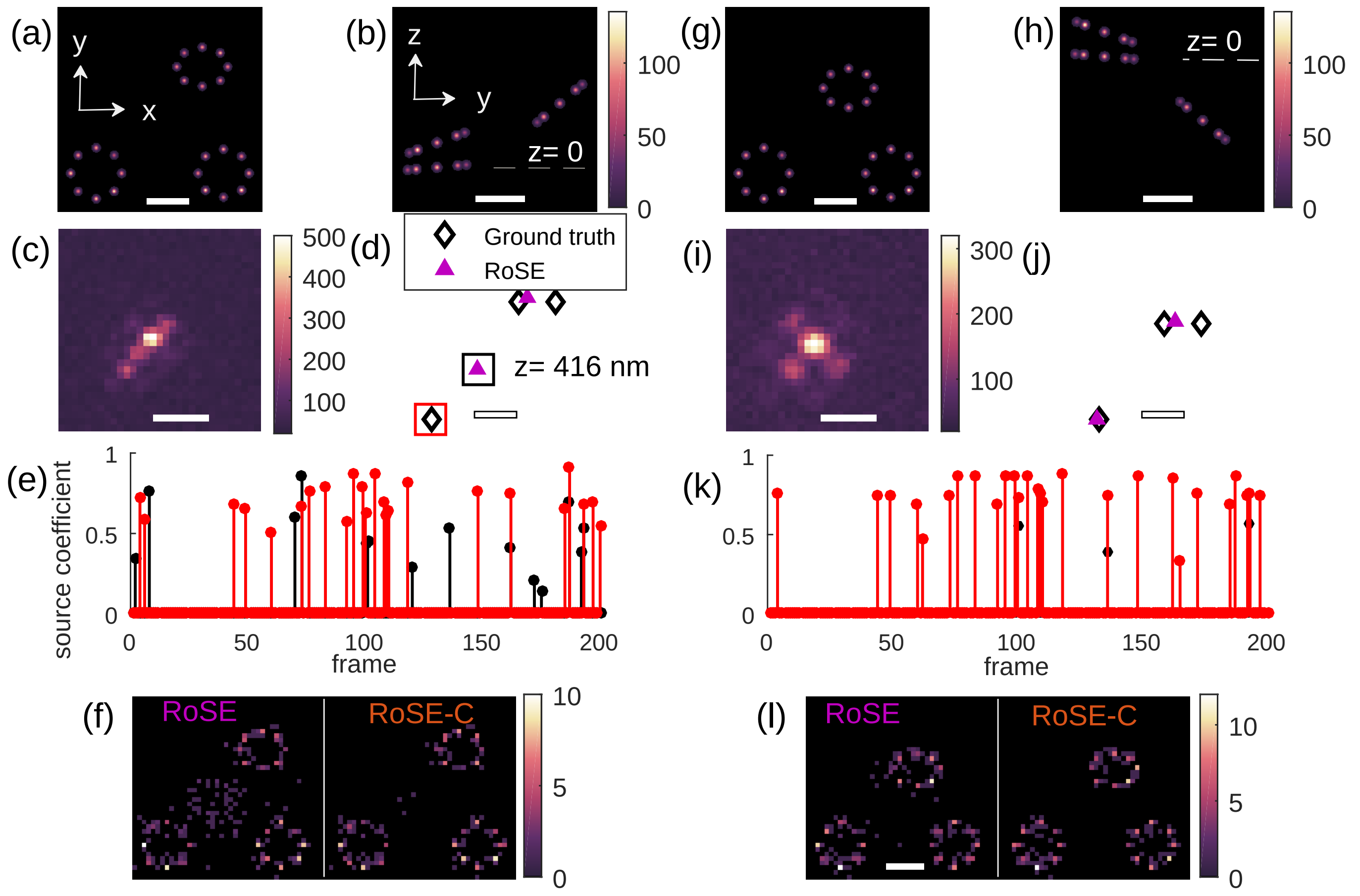

Fig. S18. Effect of sample structure (densely-packed NPCs) on recovery performance of Tetrapod PSF and the ability of RoSE-C in minimizing structural biases. (a) xy and (b) yz views of the simulated ground-truth arrangement of 3 NPCs centered at (0, 0, 0), (300, 0, 50), and (200, 200, 130) nm, each consisting of 8 labeling sites equidistantly distributed on a circle with diameter of 120 nm. The brightest pixel corresponds to the peak of a Gaussian distribution (standard deviation = 3.5 nm) multiplied by the number of blinking events. (c) A representative overlapped image, (d) corresponding ground-truth molecules (black diamonds), and recovered molecules (purple triangles) by RoSE. Color bar: (c) number of photons per 58.5×58.5 nm^2. Scale bars: (a,b) 100 nm, (c) 500 nm, and (d) 100 nm. (e) Time-trace of GradMap pixels corresponding to the (red box) ground truth and (black box) falsely-localized molecules in (d). GradMap pixel corresponding to the (red) ground-truth molecule shows larger correlation over 200 frames compared to the (black) flase one. (f) Recovered structures by RoSE and RoSE-C. For RoSE-C the autocorrelation order and threshold were set to 2 and 0.1, respectively. Color bar: (f) number of localizations per 12×12 nm^2. (g-l) Same as (a-f) but with 3 NPCs centered at (0, 0, 0), (300, 0, 50), and (200, 200, -130) nm and with different orientations. Interestingly, in (l) RoSE demonstrates a significantly-increased accuracy compared to (f). Scale bar: (l) 100 nm. In (a-l) a mean emission intensity of 3,100 photons and a mean background of 40 photons/pixel were used.

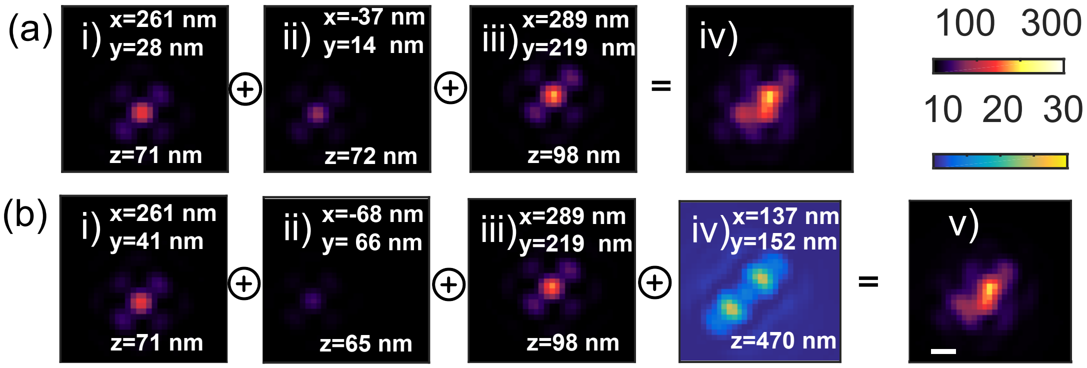

Fig. S19. An example of the tetrapod PSF degeneracy. (a(i-iii)) Simulated noiseless images of individual molecules with various positions and brightnesses and a(iv) their overlapped image. Brightnesses: i) 3,950, ii) 2,250, and iii) 5,200 photons. (b(i-iv)) Simulated noiseless images of individual molecules with various positions and brightnesses and b((v)) their overlapped image. Brightnesses: i) 1,100, ii) 3,900, iii) 4,800, and iv) 1,800 photons. Color bars: number of photons per 58.5×58.5 nm^2. Scale bar: 300 nm.

Running time of various algorithms							
dataset	number of frames	field of view (μm^2)	number of localizations		running time (s)		
			FALCON	RoSE-C	FALCON	SRRF	RoSE-C
Fig. 4	500	3.3×3.3	6,853	4,769	135	2.1	229
Fig. S13	500	3.3×3.3	12,326	9,778	178	2.5	241

Table S1. Running time of FALCON, SRRF-TRAC2, and RoSE-C. Each algorithm was run on a machine with 20 GB RAM and an Intel® Core™ i7-6700 CPU (3.40 GHz clock). Both FALCON and RoSE-C are implemented in Matlab.

REFERENCES

1. Boyd, S. & Vandenberghe, L. *Convex optimization* (Cambridge university press, 2004).
2. Tan, Z., Yang, P. & Nehorai, A. Joint sparse recovery method for compressed sensing with structured dictionary mismatches. *IEEE Trans. Signal Process.* **62**, 4997–5008 (2014).
3. Moreau, J.-J. Proximité et dualité dans un espace hilbertien. *Bull. Soc. Math. France* **93**, 273–299 (1965).
4. Beck, A. & Teboulle, M. A fast iterative shrinkage-thresholding algorithm for linear inverse problems. *SIAM J. Imaging Sci.* **2**, 183–202; 10.1137/080716542 (2009).
5. Harmany, Z. T., Marcia, R. F. & Willett, R. M. This is spiral-tap: sparse poisson intensity reconstruction algorithms—theory and practice. *IEEE Trans. Image Process.* **21**, 1084–1096 (2012).
6. Gustafsson, N. *et al.* Fast live-cell conventional fluorophore nanoscopy with ImageJ through super-resolution radial fluctuations. *Nat. Commun.* **7**, 12471; 10.1038/ncomms12471 (2016).
7. Bach, F., Jenatton, R., Mairal, J., Obozinski, G. *et al.* Optimization with sparsity-inducing penalties. *Foundations and Trends in Machine Learning* **4**, 1–106 (2012).
8. Venkataramani, V., Herrmannsdörfer, F., Heilemann, M. & Kuner, T. SuReSim: simulating localization microscopy experiments from ground truth models. *Nat. Methods* **13**, 319–321 (2016).
9. Cinlar, E. *Introduction to stochastic processes* (Courier Corporation, 2013).
10. Sage, D. *et al.* Quantitative evaluation of software packages for single-molecule localization microscopy. *Nat. Methods* **12**, 1–12 (2015).
11. Goodman, J. *Introduction to Fourier optics* (McGraw-hill, 2008).
12. Buehren, M. Functions for the rectangular assignment problem. *MATLAB Central File Exchange* (2014). http://www.mathworks.com/matlabcentral/fileexchange/6543.
13. Ehmann, N. *et al.* Quantitative super-resolution imaging of Bruchpilot distinguishes active zone states. *Nat. Commun.* **5**, 4650; 10.1038/ncomms5650 (2014).
14. Min, J. *et al.* FALCON: fast and unbiased reconstruction of high-density super-resolution microscopy data. *Sci. Rep.* **4**, 4577; 10.1038/srep04577 (2015).
15. Ovesný, M., Křížek, P., Borkovec, J., Švindrych, Z. & Hagen, G. M. ThunderSTORM: a comprehensive ImageJ plug-in for PALM and STORM data analysis and super-resolution imaging. *Bioinformatics* **30**, 2389–2390 (2014).
16. Wang, X., Chen, D., Yu, B. & Niu, H. Statistical precision in super-resolution optical fluctuation imaging. *Appl. Opt.* **55**, 7911–7916 (2016).
17. Bruckstein, A. M., Donoho, D. L. & Elad, M. From sparse solutions of systems of equations to sparse modeling of signals and images. *SIAM Rev.* **51**, 34–81 (2009).
